# Unlocking the mystery of the PD-1/PD-L1 axis: beyond the checkpoint hype

**DOI:** 10.3389/fimmu.2025.1708873

**Published:** 2025-12-04

**Authors:** Pierre-Hubert Desimpel, Pierre-Florent Petit, Benoit J Van den Eynde, Jingjing Zhu

**Affiliations:** 1 Immunity & Cancer Research Program, De Duve Institute, Brussels, Belgium; 2 Ludwig Institute for Cancer Research Brussels Branch of Human Cancer Cell Genetics, Brussels, Belgium; 3 Centre Hospitalier Universitaire (CHU) Helora association sans but lucratif (asbl), Mons, Belgium; 4 Walloon Excellence in Life Sciences and Biotechnology (WELBIO) asbl, Wavre, Belgium; 5 Department of Medicine, University of Oxford Nuffield, Oxford, United Kingdom

**Keywords:** PD-1/PD-L1, T-cell biology, immune regulation, tumor microenvironment, immuneevasion, signal transduction, post-translational regulation, protein-protein interactions

## Abstract

The discovery of the PD-1 receptor and its ligand PD-L1 revolutionized our understanding of immune regulation and, together with that of CTLA-4, allowed the development of immune checkpoint blockade, now a cornerstone of cancer therapy. Early models emphasized a simplistic view in which PD-L1 expression by tumor cells directly inhibited cytotoxic T lymphocytes through PD-1 engagement. However, recent findings reveal that this pathway is far more complex, involving multilayered regulation of PD-L1 expression, extensive post-translational modifications, and a broad spectrum of interacting partners. In addition to tumor cells, multiple immune and stromal populations, including dendritic cells, macrophages, T cells, and endothelial cells, express PD-L1 and critically shape anti-tumor immunity and therapeutic responses. Moreover, PD-L1 exerts intrinsic, non-immune functions within tumor cells, including regulation of proliferation, apoptosis resistance, and metabolic adaptation. PD-1 itself, long viewed as a T-cell-restricted inhibitory receptor, is now recognized as functionally relevant on additional cell types such as natural killer cells, myeloid cells, and even tumor cells, further diversifying its role in immune regulation and tumor biology. Together, these insights challenge the classical dogma and call for a refined view of the PD-1/PD-L1 axis that accounts for its cellular heterogeneity, molecular complexity, and bidirectional signalling. Incorporating this knowledge into clinical practice will be essential to improve patient stratification, overcome therapeutic resistance, and design innovative combination strategies to fully exploit the potential of immune checkpoint blockade.

## Introduction

In 1992, Tasuku Honjo and his team, while investigating the molecular mechanisms underlying programmed cell death, identified a gene specifically expressed in murine lymphoid cell lines undergoing apoptosis. This gene, termed *PDCD1* and encoding the protein PD-1 (Programmed Cell Death-1), belongs to the immunoglobulin superfamily and contains intracellular motifs suggestive of signalling activity (1). Although initially thought to mediate apoptosis, PD-1 was later recognised as a key immune checkpoint receptor regulating T-cell activation and peripheral tolerance.

**Figure 1:**
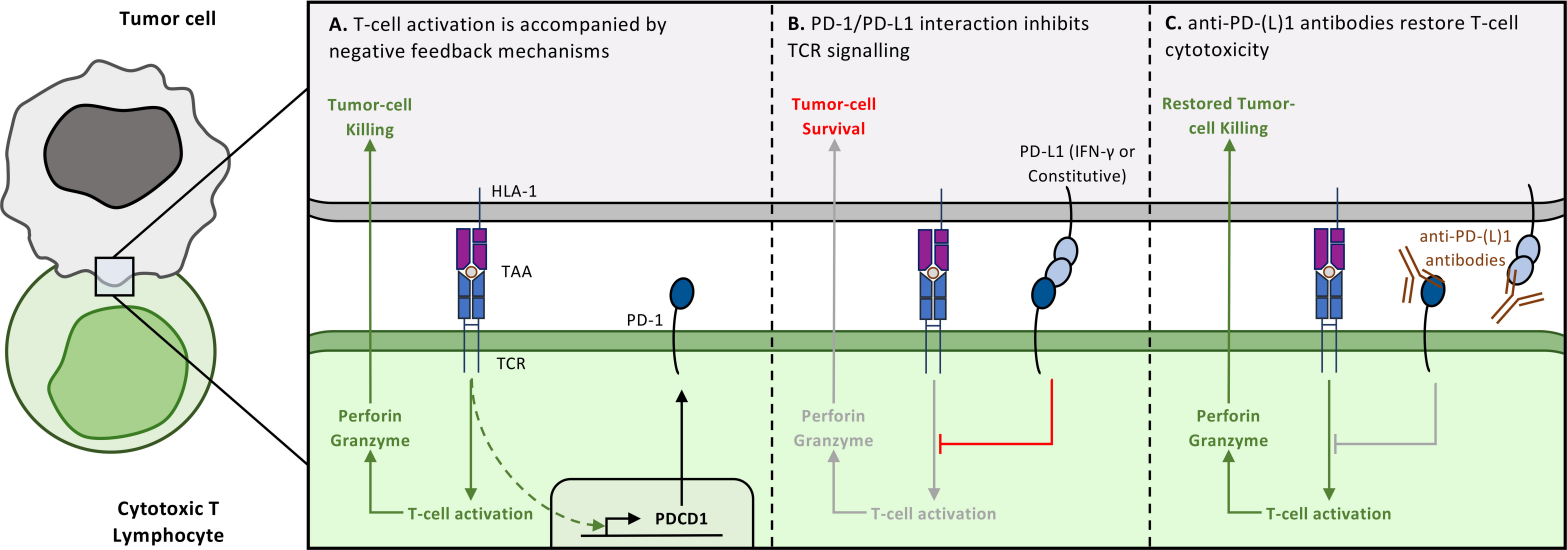
Common understanding of the PD-1/PD-L1 pathway. **(A)** PD-1 expression on Cytotoxic T Lymphocytes is the consequence of antigen-specific stimulation, in this case the recognition of a Tumor associated antigen (TAA) presented by Human Leucocyte Antigen-I (HLA-I) molecules on the surface of tumor cells. **(B)** PD-1 expression counterbalances the mechanisms associated to T-cell cytotoxicity. PD-1 interacts with PD-L1, which can virtually be expressed on all IFN-γ-stimulated cells or constitutively expressed by tumor cells. PD-1/PD-L1 interaction initiates a cascade of dephosphorylation events that ultimately inhibit TCR downstream signalling and therefore reduce T-cell cytotoxicity. **(C)** This pathway is now targeted in a wide range of cancers using monoclonal antibodies against either PD-1 or its ligand PD-L1, in order to restore anti-tumor immune responses mediated by Cytotoxic T cells.

These discoveries initiated extensive research that progressively elucidated the PD-1/PD-L1 signalling pathway, and revolutionized cancer immunotherapy. In this review, besides retracing the main milestones that enabled the translation of fundamental PD-1/PD-L1 biology into clinical benefit (**Figure 1**), we will emphasise that, despite tremendous therapeutic success of immune checkpoint blockade, the classical view of PD-1/PD-L1 pathway has become insufficient. Recent evidence highlights the complexity of their regulation, expression patterns, and molecular interactions, which we must now integrate to refine and extend the clinical efficacy of PD-1/PD-L1-targeted therapies.

## Review of a dogma: the PD-1/PD-L1 pathway

### Early days of PD-1 and PD-L1

**Figure 2:**
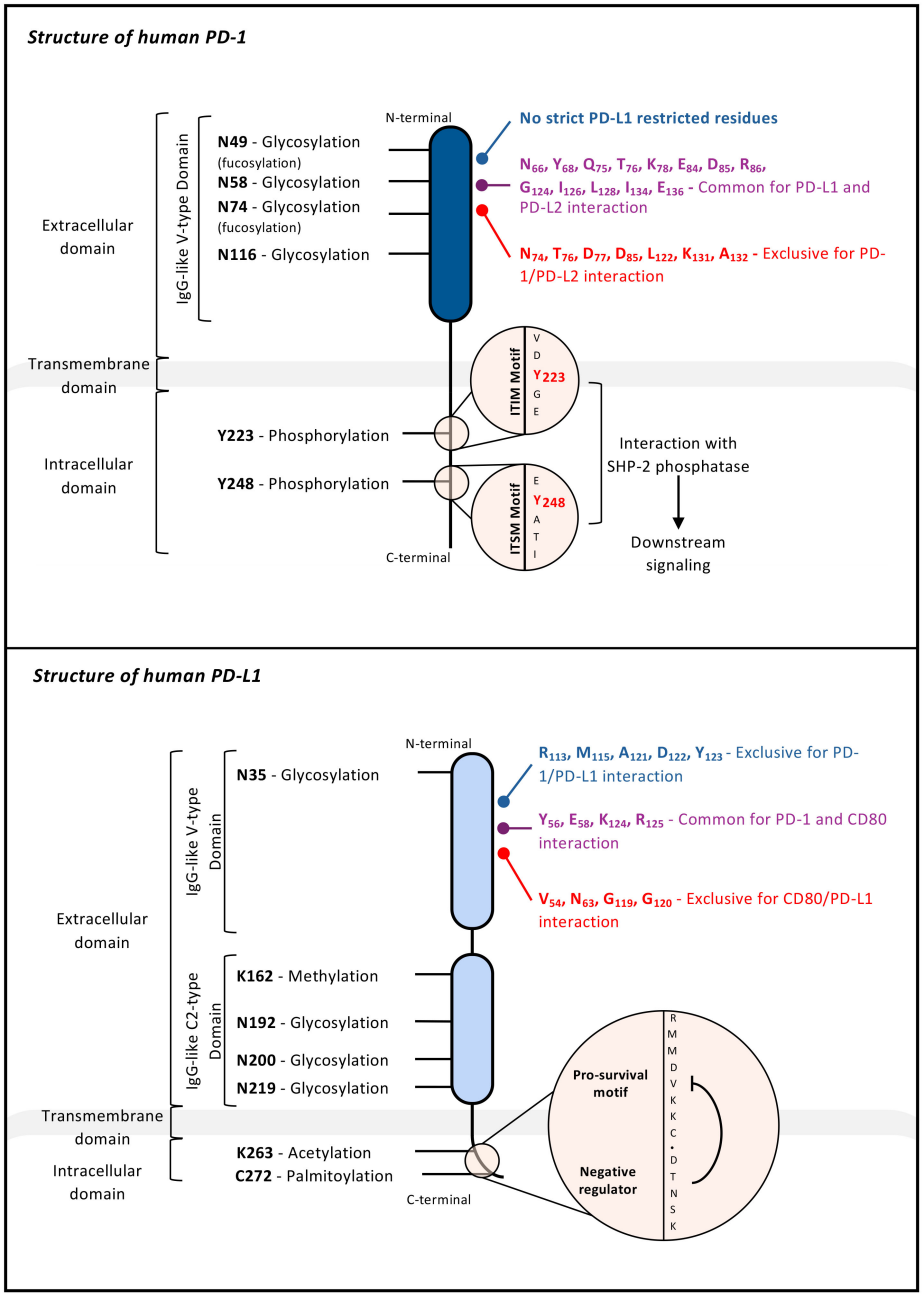
Structure of human PD-1 (top) and human PD-L1 (bottom). Schematic representation of human PD-1 and PD-L1 showing their domain architecture. Each protein comprises an N-terminal extracellular domain with a single (PD-1) or double (PD-L1) Ig-like fold, a transmembrane segment, and a C-terminal cytoplasmic tail. Both are heavily glycosylated at multiple asparagine (N) residues indicated on the diagram. The intracellular region of PD-1 contains one immunoreceptor tyrosine-based inhibitory motif (ITIM) and one immunoreceptor tyrosine-based switch motif (ITSM) required for downstream inhibitory signalling. PD-L1 harbours two conserved intracellular motifs, RMMDVKKC and DTNSK, recently linked to PD-L1-dependent resistance to type II IFN cytotoxicity. Amino acid residues critical for binding to respective partners, PD-L1 or PD-L2 (for PD-1) and PD-1 or CD80 (for PD-L1) are indicated on the extracellular domains.

During the 1990s, the group of Tasuku Honjo characterised human and murine PD-1. Human PD-1 protein (288 amino acids) shares about 60% identity with its murine counterpart. PD-1 displays the hallmarks of the immunoglobulin superfamily (**Figure 2**, Top), including an extracellular IgV domain, a transmembrane region and a cytoplasmic tail harbouring an immunoreceptor tyrosine-based inhibitory motif (ITIM) reminiscent of the motif found in other inhibitory receptors such as FcγR and KIR (2). Work by Vibhakar et al. (1997) revealed that PD-1 expression in human lymphoid cells (Jurkat and PBMCs) is not induced by apoptosis but by cellular activation and differentiation (3). Upon stimulation, PD-1 tyrosine residues become phosphorylated, supporting its role as an inhibitory signalling molecule (1).

Shortly after, was identified the first PD-1 ligand, PD-L1 (B7-H1) by the group of Lieping Chen. PD-L1 is a 290-amino acids transmembrane glycoprotein belonging to the B7 family (**Figure 2**, Bottom), with an extracellular region composed of IgV and IgC domains (4, 5). PD-L1 is abundantly expressed in the heart, lung, placenta, and skeletal muscle, and is inducible on antigen-presenting cells (APCs) following inflammatory cues (similarly to B7.1 (CD80) and B7.2 (CD86)). While initial studies diverged, Chen’s group suggested a co-stimulatory role for PD-L1, whereas Honjo’s group demonstrated T-Cell Receptor (TCR)-driven inhibition. Ultimately the accumulating data pointed toward an inhibitory function. These conflicting observations hinted early on that PD-L1 might interact with additional partners beyond PD-1.

In the early 2000s, Chen’s laboratory made a pivotal discovery: immunohistochemical analyses revealed that PD-L1 was largely absent from normal tissues, except certain macrophages, yet was highly expressed in multiple human tumors, including lung, ovarian, colon, and melanoma specimens. PD-L1 was also inducible on cancer cell lines upon IFN-γ stimulation. Functionally, PD-L1 expression rendered tumor cells resistant to Cytotoxic T Lymphocyte (CTL)-mediated killing and could even trigger CTL apoptosis, independently of PD-1 engagement (6). These findings provided the first direct link between PD-L1 expression and tumor immune evasion. Concurrently, Honjo’s team confirmed *in vivo* that PD-L1 overexpression confers a substantial growth advantage to tumor cells in a T cell- and PD-1-dependent manner (7). Together, these studies established the conceptual framework that PD-1/PD-L1 interaction suppresses anti-tumor immunity, leading to the emergence of a new therapeutic paradigm: immune checkpoint blockade (**Figure 1**).

### Targeting PD-1 and PD-L1 in cancer: first steps in the clinic

From its discovery to today, research on the PD-1/PD-L1 axis has unveiled its central role in adaptive immunity, tolerance, and disease, including autoimmunity, infection, and cancer. A decisive milestone came in 2010, when a first clinical trial revealed the striking therapeutic efficacy of PD-1/PD-L1 blockade in cancer. This trial assessed the safety and tolerance of an anti-PD-1 agent, later known as Nivolumab, on 39 treatment-refractory cancer patients (8). This report documented favourable safety outcomes, with three patients experiencing a clinical response. Two years later, a broader Phase I study involving 296 patients confirmed these results, revealing significant response rates in non-small cell lung cancer (NSCLC), melanoma, and renal cell carcinoma (RCC) (9). Concurrently, another Phase I clinical trial evaluating a PD-L1-targeting antibody on 207 patients with various cancers, including melanoma, NSCLC, and RCC, demonstrated promising results (10). In both studies, treatment responses were durable, lasting over a year and a half in half of the responsive patients. Both anti-PD-1 and anti-PD-L1 antibodies appeared to exhibit comparable efficacy and toxicity. However, it was shown early that some common tumor types were refractory to PD-1/PD-L1-targeting therapies. This remains true 10 years later for pancreatic, ovarian, prostate and most colorectal cancers.

Today, antibodies targeting the PD-1/PD-L1 axis are used in multiple indications as immunotherapy agents against cancer (Immune Checkpoint Inhibitors, ICIs). While we won’t provide an exhaustive list in this review, we refer readers to the following reference (11), which precisely outlines all indications of FDA-approved ICIs, following the guidelines of the National Comprehensive Cancer Network.

As stated in these first lines, the initial clinical success of anti-PD-1/PD-L1 antibodies was largely built on a simplified view of this pathway, primarily focusing on PD-L1 expression by tumor cells and its blockade to restore T-cell activity. In light of recent advances, it is now clear that PD-L1 has far more complex biological functions, including expression on non-tumor cells within the tumor microenvironment (TME) and involvement in intracellular signalling. These findings challenge the initial dogma and call for refined clinical strategies. To fully harness the therapeutic potential of PD-1/PD-L1 inhibitors, it is essential that these nuances be integrated into daily clinical practice, informing patient selection, therapeutic combinations, and resistance mechanisms. Accumulating evidence has revealed that PD-L1 is far more than a passive inhibitory molecule. The following sections will explore this deeper understanding of PD-L1 biology and its potential clinical implications.

## What do we really know about programmed cell death 1 ligand 1?

### Regulatory mechanisms of PD-L1 expression

**Figure 3:**
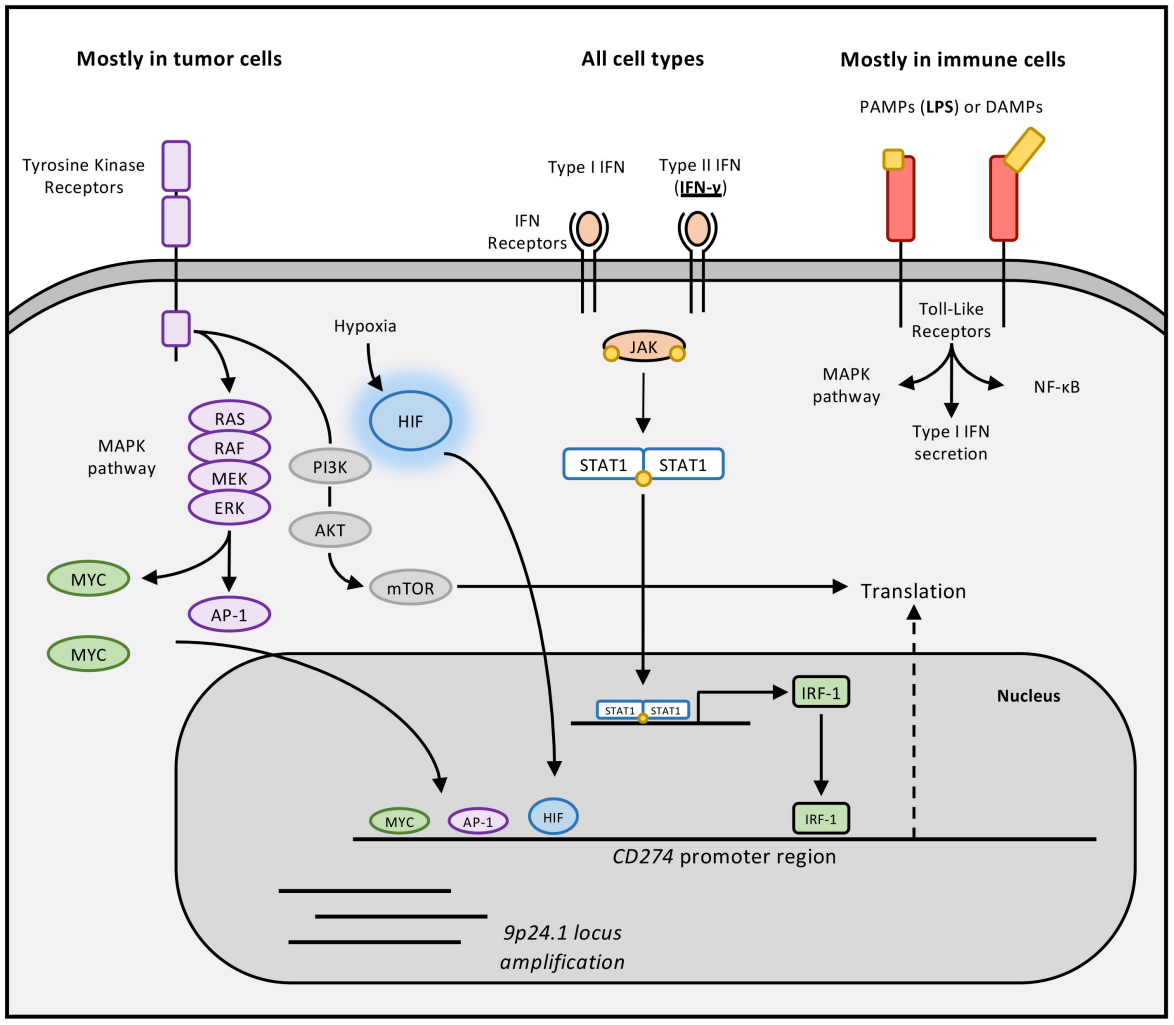
Regulatory mechanisms of PD-L1 expression. PD-L1 expression can be mediated by various pathways. In this figure, we make a distinction between the canonical pathway that is IFN-γ-dependent, and pathways that are restricted to immune cells or restricted to tumor cells.

There are five major intracellular signalling pathways that can influence the transcription of the gene encoding PD-L1 (namely *CD274*): JAK/STAT/IRF-1; NF-κB; MAPK; PI3K/AKT; and HIF-1α (**Figure 3**). It is noteworthy that the activation of these signalling pathways depends on a multitude of extrinsic and intrinsic factors (cell type, primary messenger, mutations, cellular microenvironment, etc.) (12).

When PD-L1 was discovered, it was quickly identified that its expression was regulated by Interferon-γ (IFN-γ). In the early 2000s, Tasuku Honjo’s team demonstrated that monocytes isolated from PBMCs and treated with IFN-γ significantly increased *CD274* transcription (5). This was also true for keratinocytes treated with IFN-γ. In 2006, the mechanism by which IFN-γ increases PD-L1 expression was identified (13). Cloning and characterizing the promoter upstream of the *CD274* gene revealed the presence of several sequence motifs corresponding to binding sites for different transcription factors. Among them were two for Interferon Regulatory Factor-1 (IRF-1). Presumably, by binding to its receptor, IFN-γ allows the phosphorylation and activation of JAK1 and JAK2, which in turn phosphorylate STAT1 and STAT3. The STATs induce the expression of IRF-1, leading to increased PD-L1 expression. It is worth noting that there are basal levels of IRF-1 expression, which could be responsible for the rapid effect of IFN-γ on PD-L1 expression. In practice, any cell exposed to inflammation in the presence of type II IFN can express PD-L1. Other inflammatory cytokines, including type I interferons, interleukins and TNF-α, can also upregulate PD-L1 through similar signalling pathways (14).

As mentioned earlier, the promoter of *CD274* has other sequence motifs allowing the binding of other transcription factors. Nuclear factor-κB (NF-κB) binds to three motifs in the *CD274* promoter to activate transcription. This regulation occurs when monocytes encounter inflammatory cues such as pathogen-or damage-associated molecular patterns (PAMPs, DAMPs). In this situation, these molecular determinants bind to Toll-Like Receptors (TLR), whose activation ultimately leads to NF-κB activation (15). However, PD-L1 expression due to TLR activation does not solely depend on the NF-κB transcription factor. In 2008, Qian et al. demonstrated that ERK and JNK inhibitors were able to drastically attenuate the increase in PD-L1 expression following TLR4 signalling activation in bladder cancer cells (16). PD-L1 induction also relies on MAPK and PI3K-AKT activation. *CD274* promoter contains binding sites for Myc and c-Jun, linking oncogenic signalling to PD-L1 transcription. Although present physiologically in normal cells, these signalling pathways leading to PD-L1 expression have primarily attracted attention in tumor cells. Indeed, in these cells, a strong and constitutive expression of PD-L1 is not uncommon (17). These effects result from dysregulated MAPK, PI3K/AKT and, in some cases, JAK/STAT/IRF-1 signalling (18). Such dysregulation often reflects sustained activation of tyrosine kinase receptors like EGFR or oncogenic mutations in downstream components. Nevertheless, the debate persists as to whether PD-L1 expression in these contexts is truly a “desired” effect by tumor cells to promote their proliferation, a form of innate and/or adaptive resistance to anti-tumor immunity, or a fortuitous consequence of multiple dysfunctions occurring within tumor cells.

In the context of cancer, there is a mechanism leading to the induction of *CD274* transcription that can be considered specific to the stressful environment characteristic of tumors, namely hypoxia. Hypoxia is characterized by a decrease in oxygen supply due to the scarcity of blood vessels within tumor masses. Hypoxia is a hallmark of cancer. At the molecular level, oxygen depletion stabilizes Hypoxia-Inducible Factor-1α (HIF-1α), a transcription factor normally degraded under normoxia. HIF-1α allows cells to adapt to low oxygen. About a decade ago, Barsoum et al. demonstrated that tumor cells in culture exposed to hypoxia increased PD-L1 expression (19). Chip-sequencing revealed that HIF-1α interacts with the gene encoding PD-L1 by binding to a Hypoxia Response Element located in the intron between exons 4 and 5 of the gene. Hypoxia upregulates PD-L1 not only in tumor cells but also in stromal components of the TME (20).

Lastly, PD-L1 expression can also result from increased PD-L1 gene copy numbers (21). Classical Hodgkin Lymphoma (cHL) represents a striking example, as amplification of the 9p24.1 chromosomal region, harboring PD-L1, PD-L2, and JAK2, is a hallmark of the disease (22). These genetic alterations are believed to contribute to the remarkable clinical efficacy of PD-1 blockade in this malignancy. Indeed, patients with relapsed or refractory cHL typically experience some of the highest response rates reported for PD-1/PD-L1 inhibitors, with even single-dose nivolumab showing exceptional activity (23, 24).

### How can we integrate the understanding of regulatory mechanisms leading to PD-L1 expression in cancer patient?

Distinguishing oncogenic from immune-driven PD-L1 can help predict responses to PD-1/PD-L1-targeting immunotherapies. For instance, immune-driven PD-L1 expression can indicate an active immune response in patients, which may predict a higher likelihood of clinical success with immunotherapy (25, 26). Additionally, the signalling pathways responsible for oncogenic PD-L1 expression are often involved in tumor-cell proliferation and survival. Combining PD-1/PD-L1 blockade with targeted inhibitors that reduce PD-L1 expression may enhance efficacy, though clinical proof is still limited (27, 28). Moreover, understanding the mechanisms behind PD-L1 expression can shed light on the development of resistance to immunotherapy and how tumor cells continue to evade the immune system (29, 30). In cases of constitutive PD-L1 expression, its levels may remain elevated even in the presence of immune attack following immunotherapy, which supports the rationale for combination therapies. On the other hand, IFN-γ-induced PD-L1 expression is often transient and reflects immune pressure. Understanding these nuances can inform more personalized and effective cancer treatment strategies.

### Post-translational regulation of PD-L1

**Figure 4:**
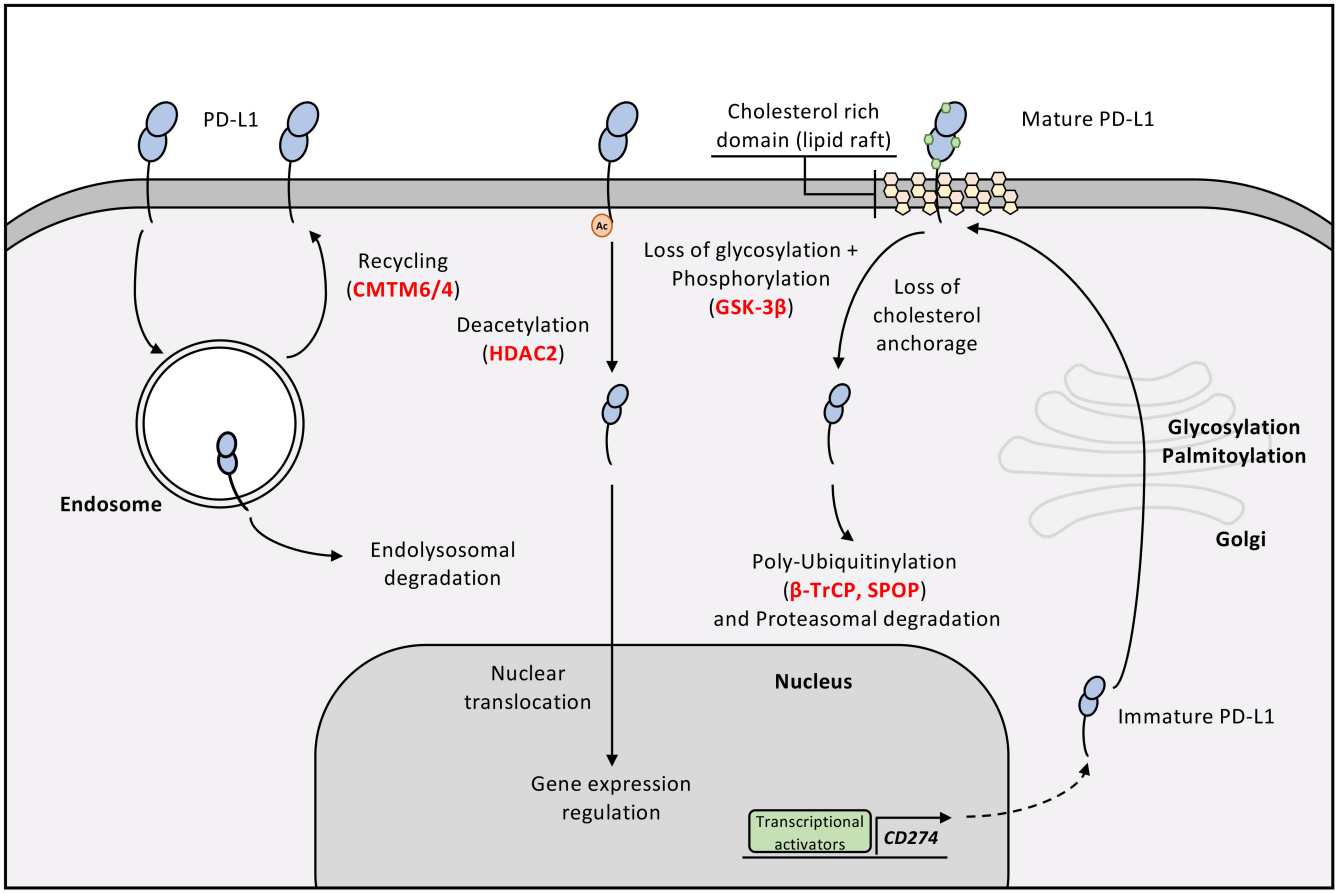
Post-translational modifications of PD-L1. The main post-translational modification of PD-L1 is the addition of sugar moities on its extracellular domain, highlighted by green dots on mature PD-L1. Beside glycosylation, PD-L1 stability at the plasma membrane is maintained by several other factors: its anchorage to cholesterol rich domains, its acetylation pattern, but also its interaction with CMTM6. If one of these factors is abrogated, PD-L1 is rapidly degraded, either by endosomal degradation or by proteasomal degradation. Importantly, loss of acetylation drives PD-L1 towards the nuclei where it can act as transcription co-factor.

PD-L1 gene transcription involves various signals, and the newly synthesized protein undergoes precise post-translational regulations. Post-translational modifications (PTMs), while leaving PD-L1’s amino acid sequence unchanged, commonly modulate its stability on the cell membrane and alter its subcellular localization (**Figure 4**). While glycosylation has been the most extensively characterized and functionally validated PTM (with potential implication in the use of PD-L1 as a biomarker, as seen later), palmitoylation is also supported by mechanistic studies and therefore reported as key regulator of PD-L1 at the plasma membrane. Acetylation and methylation of PD-L1 have been described more recently. Although conceptually compelling, their physiological relevance in human tumors remains to be confirmed.

PD-L1 is a glycoprotein with four N-glycosylation sites in its extracellular domain. Substituting three of these glycosylation motifs significantly decreases PD-L1 stability on cell surfaces, leading to rapid degradation by the proteasome (31). Mechanistically, deglycosylation of PD-L1 unveils an interaction and phosphorylation motif for GSK-3β that has been conserved throughout evolution. The subsequent phosphorylation of PD-L1 by GSK-3β exposes a degradation motif (DEGRON), facilitating its recognition by an E3-Ubiquitin ligase (β-TrCP). PD-L1 is then polyubiquitinylated and degraded by the 26S proteasome. How deglycosylation of residues in the extracellular domain would expose them to intracellular GSK-3β and β-TrCP remains unclear. Although internalization or intracellular trafficking of PD-L1 could theoretically permit such access, this was not addressed in the study and represents a limitation of the proposed mechanism. In tumor cells, PD-L1 glycosylation is promoted by EGF receptor (EGFR) activation, linking oncogenic signalling pathways to PD-L1 expression in tumors (32).

Additionally, the intracellular domain of PD-L1 undergoes palmitoylation, a PTM regulating protein localization and function. Recent research revealed that modulating palmitoylation can stabilize PD-L1 (33). Palmitoylation occurs at cysteine 272, and its addition by DHHC3 acyltransferase protects PD-L1 from monoubiquitinylation and lysosomal degradation. PD-L1 palmitoylation was recently shown to impact its membrane localization and orientation, thereby regulating its binding to PD-1 (34). Indeed, inhibiting PD-L1 palmitoylation reduced its lipid raft affinity, causing a conformational reorientation of its extracellular domain that sterically limits PD-1 engagement.

Acetylation at lysine 263, catalysed by p300 and reversed by HDAC2, also regulates PD-L1 (35). Surprisingly, deacetylated PD-L1 shifts to the cell nucleus via clathrin-dependent endocytosis. Nuclear PD-L1 acts as a cofactor for transcription factors, positively regulating genes involved in inflammation and anti-tumor immune responses.

A recent study examined how PD-L1 PTMs affect PD-1 binding (36). In NSCLC trials, PD-L1 expression did not always correlate with response, suggesting that certain PTMs may impair PD-1/PD-L1 engagement and limit treatment efficacy. The author hypothesized that PTMs hinder PD-1/PD-L1 interaction, which could explain why treatment is ineffective despite the expression of PD-L1. Inhibitors targeting methylation, glycosylation, acetylation, and phosphorylation were tested, revealing that while deglycosylation disrupts PD-1/PD-L1 interaction, demethylation has opposite effects. Methylation at lysine 162, catalysed by the methyltransferase SETD7, was identified as a key modification.

In 2017, Mezzadra et al. used a genome-wide genetic screen to identify novel regulators of PD-L1 (37). Their innovative approach pinpointed CMTM6 (CKLF-like MARVEL transmembrane domain-containing proteins 6) as a key player, with subsequent validation experiments confirming its impact on PD-L1 protein levels. CMTM6 stabilizes PD-L1 by preventing its ubiquitination and lysosomal degradation after endocytosis, thereby forcing its recycling to the cell surface. Its homolog CMTM4 shares this function. In a complementary investigation, Burr et al. explored the functional implications of CMTM6 in maintaining PD-L1 expression and its effects on anti-tumor immunity (38). They demonstrated that the interaction of CMTM6 with PD-L1 is essential for sustaining surface PD-L1 levels on tumor cells, which in turn enables these cells to evade immune surveillance by inhibiting T-cell activity.

Finally, PD-L1 stability also depends on plasma-membrane cholesterol. Wang et al. showed that PD-L1 contains two cholesterol-recognition amino acid consensus (CRAC) motifs, which promote its association with cholesterol-rich membrane domains (lipid rafts) (39). Of note, specific depletion of plasma membrane cholesterol using methyl-β-cyclodextrin or statins such as Simavastin lead to decreased plasma membrane PD-L1 expression and total PD-L1 expression, suggesting that loss of PD-L1/cholesterol interaction leads to PD-L1 degradation. PD-L2, the other ligand of PD-1 and homolog of PD-L1 is also stabilized at the plasma membrane by cholesterol, though through a different mechanism as compared to PD-L1 (40).

### Why should we care about PD-L1 post-translational modifications?

The interest of all these PTMs regulating PD-L1’s stability on the cell membrane relies in their potential diagnostic and therapeutic applications. Considering that PD-L1 glycosylation is strongly influenced by EGFR signalling and other tyrosine kinases (TKs), TK inhibitors can synergize with PD-1 blockade to restore anti-tumor immunity. Combinations directly targeting EGFR and immune checkpoints have failed to demonstrate clinical benefit in phase III trials such as KEYNOTE-789 (41) and CheckMate-722 (42) and may also induce severe toxicity (43). However, in a key subgroup of EGFR-mutated NSCLC patients, regimens combining ICIs with anti-VEGF agents and chemotherapy have yielded progression-free and overall survival improvements (44). This suggests that targeting and shaping the TME is key to observe objective response in patients (44–46).

Similarly, HDAC inhibitors (HDACi) block PD-L1 deacetylation and prevent its nuclear entry, thereby limiting PD-L1-mediated activation of inflammatory and immune-response genes. Moreover, HDACi have been shown to unleash the expression of various T-cell chemokines in the TME. Together, in pre-clinical models, these effects contribute to a more robust response to immunotherapies targeting PD-1/PD-L1 (47). Despite promising preclinical data and early-phase combinations (e.g. HDACi + anti-PD-1 in solid tumors) showing safety and clinical benefit to patients (48, 49), this combination therapy needs further translational exploration as clinical trials are still ongoing.

In a similar vein, the use of palmitoylation inhibitors appears to revive anti-tumor immunity and enhance the infiltration of tumors by CTLs (33). On the other hand, PD-L1 methylation status could serve as a potent biomarker to predict which patients’ tumors are likely to experience the PD-1/PD-L1 interaction, thereby anticipating those who may or may not respond to PD-1/PD-L1 axis-targeting treatment. Currently, these novel concepts are not yet incorporated into clinical routine but provide a broad range of options to enhance anti-PD-1/PD-L1 immunotherapy and offer it to patients who are most likely to benefit from it.

Concomitantly, studies investigating the interaction between PD-L1 and CMTM6 shed the light on the therapeutic potential of targeting CMTM6 along with PD-L1. Indeed, it has been shown that knockdown of CMTM6 in tumor models led to a significant reduction in PD-L1 levels, thereby enhancing T-cell-mediated anti-tumor responses (38).

Lastly, PD-L1 stabilization by cholesterol is of particular interest considering the increasing body of evidence of the potential benefit of using statins in cancer patients (50). Statins could reduce PD-L1-mediated immune evasion and help restore anti-tumor immunity. Clinically, multiple retrospective cohorts and meta-analyses have associated concomitant statin use with improved outcomes among patients receiving ICIs, including higher objective response rates and prolonged progression-free survival (51–53). However, results remain heterogeneous across tumor types and study designs, and a recent pharmacovigilance analysis reported a higher risk of immune-related adverse events among statin users (54). To date, no randomized trial has prospectively tested statins with ICIs or incorporated PD-L1-cholesterol biomarkers for patient selection. This strategy therefore remains hypothetical and requires dedicated biomarker-driven studies.

### Redefining PD-L1 interactions at the cell surface

**Figure 5:**
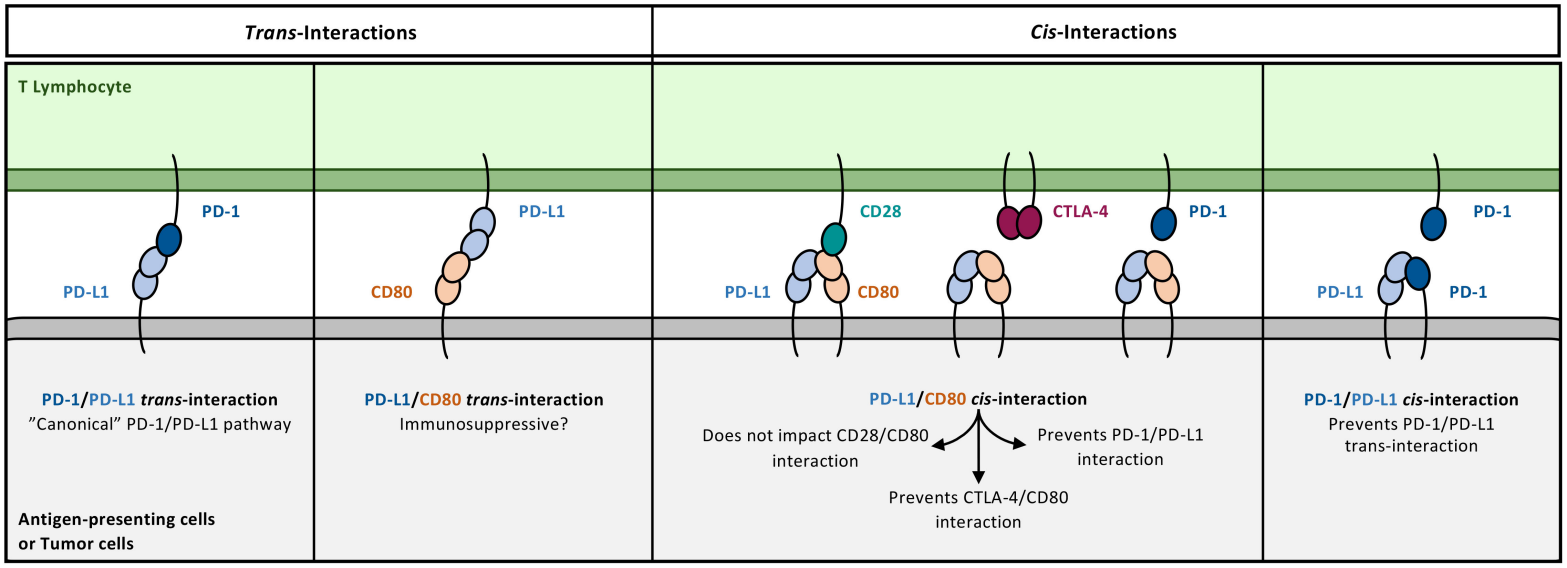
PD-L1 interactome. Since the identification of PD-L1/CD80 interaction, the understanding of the PD-1/PD-L1 pathway underwent profound adjustment. The cis-PD-L1/CD80 abrogates both PD-1/PD-L1 and CTLA-4/CD80 interactions, while impact on CD28/CD80 interaction has not been found. Consequently, targeting PD-L1/CD80 interaction has multiple impacts, either in the context of autoimmune diseases or in cancer. While anti-PD-L1 antibodies known to block both PD-1/PD-L1 and PD-L1/CD80 interaction restore CTLA-4/CD80 interaction, anti-CD80 antibodies specifically designed to block PD-L1 interaction with CD80 allow PD-L1 to bind PD-1 and induce immunosuppressive effects. Besides cis-PD-L1/CD80 interaction, PD-L1 has been shown to be involved in cis-PD-1/PD-L1 interaction and trans-PD-L1/CD80 interaction, although their physiological relevance is not well established so far.

In the early reports describing PD-L1 as a ligand for PD-1, it was quickly observed that the latter was not its sole interaction partner (**Figure 5**). In 2007, Gordon J. Freeman’s team highlighted PD-L1’s binding to CD80, conducting the first study characterizing this interaction (55). Through various *in vitro* tests using recombinant proteins, they demonstrated that PD-L1/CD80 heterodimers have lower affinity than PD-1/PD-L1 or CTLA-4/CD80 dimers but stronger affinity than CD28/CD80 dimers. PD-L1 residues involved in the PD-L1/CD80 interaction partially overlap with those in PD-1/PD-L1 and CTLA-4/CD80 interactions.

The question of how PD-L1 and CD80 interact with each other was debated for years in the literature, with both *cis* (on the surface of the same cell) and *trans* (between different cells) interactions described. Only in the late 2010s did a consensus emerge, favouring *cis*-PD-L1/CD80 interaction, forming surface heterodimers on cells expressing both proteins. It’s important to note that, to date, no study formally excludes the existence and significance of *trans* interaction.

In 2018 and 2019, three studies emphasized the PD-L1/CD80 *cis* interaction. The first, from the team that initially identified this interaction, used the NanoBiT proximity assay system in transfected HEK293H cells to demonstrate that the conformational characteristics of PD-L1 and CD80 favoured their interaction on the surface of the same cell in parallel orientations (56). This interaction competes with *trans*-PD-1/PD-L1 interaction, utilising similar PD-L1 amino acids. These results aligned with an earlier report in The Journal of Immunology, where Haile et al. showed that coexpression of PD-L1 and CD80 by tumor cells led to reduced detection of PD-L1 on the cell surface and eliminated immunosuppressive effects associated with PD-1/PD-L1 interaction (57). In 2019, two other studies approached the question of PD-L1/CD80 *cis* interaction from distinct perspectives. Sugiura et al. demonstrated that coexpression of PD-L1 and CD80 by primary mature dendritic cells (DCs) abrogated PD-1-dependent inhibition of murine CD4^+^ and CD8^+^ T cells (58). *In vivo*, in mice, using mutants of CD80 and PD-L1 lacking their interaction capability (thus releasing PD-L1 and restoring its interaction with PD-1), it was shown that tumors, initially responsive to vaccination, eventually escaped the immune system. Concomitantly, in a murine model of autoimmune encephalomyelitis, animals presented a milder phenotype in the absence of *cis*-PD-L1/CD80 interaction. Furthermore, Zhao et al. delved into the consequences of *cis*-PD-L1/CD80 interaction on other interactions involving CD80, namely with CD28 and CTLA-4, using various artificial *in vitro* reconstitution models and transfected cells, along with blocking antibodies (59). CD80 interacts with CD28 as monomers, while CTLA-4 and CD80 form lattices by interacting as dimers. PD-L1 disrupts CD80 homodimerization and thus its interaction with CTLA-4. Since homodimerization is not necessary for CD28/CD80 interaction, it is preserved in the presence of PD-L1.

Some reports are still trying to demonstrate the relevance of the interaction between PD-L1 and CD80 in *trans*. In a recent study, Zhang et al. used a murine model combining the implantation of wild-type, *CD274^KO^*, or *CD80^KO^* tumor cells, in wild-type, *CD274^KO^*, or *CD80^KO^* mice, to modulate the PD-L1/CD80 interaction in *cis* and *trans* (60). They demonstrated that in settings where only the *trans*-PD-L1/CD80 interaction can exist (implantation of *CD274^KO^* tumor cells in *CD80^KO^* mice), blocking this interaction, without interfering with the PD-1/PD-L1 interaction, is sufficient to increase anti-tumor immunity. Thus, the interaction between PD-L1 on T cells and CD80 on tumor cells or APCs appears to have immunosuppressive effects against T cells. It seems therefore that the *trans* interaction between PD-L1 and CD80 has opposite effects to the *cis* interaction. Nevertheless, in models where both modalities of interaction coexist, the net result of blocking the PD-L1/CD80 interaction *in vivo* is an increased T cell-mediated anti-tumor immunity (60).

It is worth noting that immunotherapies targeting the PD-1/PD-L1 axis can affect both T-cell interactions with APCs and with tumor cells. However, since CD80 is predominantly expressed by professional APCs and rarely by tumor cells, the interplay between the PD-1/PD-L1 and CD28/CTLA-4/CD80 pathways is likely to be more relevant in the context of T cell-APC interactions. As we will discuss later, DCs, being both strong expressers of PD-L1 and the main source of CD80, are increasingly recognized as key regulators of anti-tumor immunity in response to PD-1/PD-L1 blockade.

Another layer of complexity arises from PD-1/PD-L1 coexpression. In 2018, Enfui Hui and colleagues showed that some tumor cells and tumor-infiltrating APCs in NSCLC coexpress both PD-1 and PD-L1 (61). Using *in vitro* reconstitution models, the authors showed that PD-1 and PD-L1 interact in *cis* at the cell surface of coexpressing cells. This interaction prevents the canonical *trans* interaction and thus abrogates the immunosuppressive effects of PD-L1 expressed by tumor cells and APCs towards PD-1-expressing cells. However, this report leaves many questions unanswered: firstly, the authors did not assess the consequence of such interaction on the *in vivo* immune response. Secondly, it overlooks the fact that activated T cells also coexpress PD-1 and PD-L1 (62): does a *cis* interaction of these two proteins activate the downstream signalling pathway of PD-1? Investigating the roles of the *cis*-PD-1/PD-L1 interaction on T cells appears important in the context of PD-1/PD-L1-targeting immunotherapies.

### Can we use the knowledge about interactions regulating PD-L1 in clinical contexts?

Considering the functional importance and potential implications of the *cis*-PD-L1/CD80 interaction in pathologies such as cancer and autoimmune diseases, several recent studies have evaluated the possible therapeutic benefit of disrupting this interaction to modulate the balance of inhibitory and coactivating signals provided to T cells. Firstly, Zhao et al. demonstrated that the use of certain antibodies targeting PD-L1, known to block the *trans*-PD-1/PD-L1 interaction, also block the heterodimerization of PD-L1 and CD80 in *cis* (59). Consequently, CD80 molecules can form homodimers, which favours their interaction with CTLA-4 in *trans* and enhancing inhibitory signalling. The authors propose that combining antibodies targeting PD-L1 (to disrupt both *trans* PD-1/PD-L1 and *cis* PD-L1/CD80 interactions) with anti-CTLA-4 antibodies might further enhance T-cell reinvigoration in cancer patients. To date, no clinical trial has directly evaluated the therapeutic benefit of such a combination. Mayoux et al. provide additional support for this strategy, showing that while it remains formally undemonstrated whether the *cis*-PD-L1/CD80 interaction sterically blocks the *trans*-CD28/CD80 interaction, disrupting the *cis*-PD-L1/CD80 complex does enhance CD28 co-stimulation, suggesting functional interference between the two (63).

Additionally, in 2022, Sugiura et al. developed an anti-CD80 antibody capable of disrupting *cis*-PD-L1/CD80 heterodimers while having no impact on classical CD28/CD80 and CTLA-4/CD80 interactions. Consequently, PD-L1 becomes available for *trans*-PD-1/PD-L1 interactions and provides PD-1-associated inhibitory signals (64). This approach could be considered in the context of autoimmune diseases to alleviate symptoms related to T-cell overactivation.

### Importance of PD-L1 expression by non-tumor cells in cancer

Cancer immunology research has extensively focused on the significance of PD-L1 expression by tumor cells and its role in anti-tumor immune responses. Over the past few years, an increasing number of studies have indicated that the expression of PD-L1 on tumor cells is not always essential for the response to PD-1/PD-L1 interaction blockade. Instead, it has been shown that PD-L1 expression by cells notably belonging to the hematopoietic compartment, particularly myeloid cells, greatly contributes to the immunosuppression induced by PD-L1.

In 2017, Lau et al. conducted a pivotal study demonstrating the significance of PD-L1 expression on both tumor cells and host immune cells in mediating immunosuppression within the TME (65). By employing mouse models, they showed that PD-L1 expression on both tumor and host cells is necessary to suppress anti-tumor immunity effectively. Using FTY720, the authors showed that blocking T-cell egress from secondary lymphoid organs abolished the response to anti-PD-L1. This indicates that efficacy relies not only on reinvigorating pre-existing Tumor-Infiltrating Lymphocytes (TILs) but also on the continuous recruitment of newly primed lymphocytes from tumor-draining lymph nodes. Building on this foundation, Tang et al. showed that PD-L1 is often higher on myeloid than on tumor cells in the TME and tumor-draining lymph nodes, and is essential for tumor regression after PD-L1 blockade (66). Within the myeloid compartment, DCs likely play a central role in PD-L1-mediated immunosuppression. Although Tumor-Associated Macrophages (TAMs) are more abundant in the TME, DCs often express higher PD-L1 levels. The significant role of DCs in the PD-L1 pathway is highlighted by the fact that selectively deleting PD-L1-expressing macrophages does not affect the response to anti-PD-L1 monoclonal antibodies, whereas the loss of PD-L1^+^ DCs abolishes their therapeutic effects (67–69).

The role of PD-L1 expression on TAMs remains debated in the literature. Intuitively and based on several mouse studies, PD-L1 expressed by TAMs is thought to contribute to the inhibition of anti-tumor immune responses, similarly to DCs. However, clinical data do not fully support this view. In several cohorts, PD-L1^+^ TAMs correlate with improved overall survival in lung, breast and liver cancers. In a recent study combining single-cell RNA sequencing and multiplex immunofluorescence analyses of human breast tumors samples, Wang et al. demonstrated that PD-L1^+^ TAMs exhibit more mature and activated transcriptomic profile compared to their PD-L1^–^ counterparts. PD-L1^+^ TAMs were spatially found in closer vicinity to T cells, whereas PD-L1^–^ TAMs were preferentially localised near tumor cells (70). Functional *in vitro* assays further revealed that PD-L1^+^ TAMs enhanced CD8^+^ T-cell proliferation and cytotoxicity. It appears that the immunostimulatory functions of PD-L1^+^ TAMs is not attributed to the engagement of the PD-1/PD-L1 pathway. One possible explanation can come from the dual expression of PD-L1 and CD80, which may rather limit the CTLA-4 axis while preserving CD28 co-stimulation in T cells, as we discussed earlier. Yet, PD-L1 expression by TAMs has not been clearly correlated with clinical response to ICIs, but this study paves the way for further evaluation of their potential predictive value in this context.

Besides tumor cells and myeloid cells, other cells within the TME express PD-L1 and must be considered in our understanding of the PD-L1 biology in cancer. Among those cells, it is worth discussing the expression of PD-L1 by T cells and endothelial cells.

The role of PD-L1 expression on T cells is not yet fully understood under physiological conditions, and it is still debated whether it has a positive or a detrimental impact on their functions (62, 71–73). It is likely that PD-L1 functions on T cells depend on their activation status and the context in which they are studied. However, a report published by Diskin et al. evaluated the role of PD-L1 on T cells in mouse tumor models of pancreatic ductal adenocarcinoma (62). Consistently with previous studies, the report showed that PD-L1 is expressed on T cells upon antigen stimulation and favoured by inflammatory cues. *In vivo* experiments using adoptive cell transfer of genetically PD-L1-deleted T cells demonstrated that T cells-PD-L1 restricts anti-tumor immunity. While PD-L1 ligation on T cells seems to inhibit their cytotoxic activity, it also impacts neighbouring cells in the TME by inhibiting other PD-1-expressing T cells and influencing macrophages differentiation towards an anti-inflammatory phenotype. These findings were further supported by a recent study that used Cytometry by Time-of-Flight and imaging Mass Cytometry to investigate the CD8^+^ T cell compartment in lung cancer patients (74). This study revealed the presence of PD-L1^+^CD8^+^ T cells within tumor lesions, located closely to PD-1^+^CD8^+^ T cells. *In vitro* coculture experiments demonstrated that PD-L1-expressing T cells can inhibit the proliferation of neighbouring T cells, as well as the production of IFN-γ and TNF-α.

IFN-γ-induced PD-L1 on endothelial cells was early identified as a potent inhibitor of T-cell cytotoxic functions (75, 76). Two 2020 reports showed that tumor-endothelial cells can express PD-L1 and consequently negatively regulate the function and proliferation of CTLs and lead to their apoptosis (77, 78). In mice, *in vivo* deletion of PD-L1 on endothelial cells provided potent anti-tumor immunity as CD8^+^ T-cell apoptosis was reduced. In lung, kidney, and colon cancer patients, PD-L1 expression by tumor endothelial cells was markedly increased compared to healthy tissues. This was correlated to poor effector T-cell infiltration within the tumors and increased proportion of regulatory T cells (Tregs). More recently, it was proposed that lymphatic endothelial cells (LECs) do also express PD-L1 (79–81). Surprisingly, PD-L1 expressed on LECs not only promoted the trans-endothelial migration of induced Tregs (iTregs) but also signalled within LECs to support endothelial architecture (79). Mechanistically, this effect was mediated via PD-1/PD-L1 interaction, likely involved in iTreg migration, and PD-L1/CD80 interactions, potentially contributing to effector T-cell migration, although the precise expression patterns of PD-1 and CD80 on these T cell subsets remain to be fully clarified. Concerning effector T cells, however, the available literature remains less conclusive. Although PD-L1 expression on LECs has been reported to facilitate T-cell trans-endothelial migration, other studies have highlighted its role in maintaining peripheral tolerance. This was further supported by the work of Cousin et al., in which LEC-specific deletion of PD-L1 in murine tumor models enhanced tumor-specific CD8^+^ T-cell infiltration within the TME and modestly reduced tumor growth (80). These findings indicate that the immunological role of lymphatic PD-L1 is context-dependent, likely influenced by its expression level, spatial distribution, and tissue-specific cues. Direct clinical data remain scarce, but recent spatial transcriptomic analyses have identified tumor-associated high endothelial venules (HEVs) as key entry gates for effector T cells into the TME (82). Moreover, HEVs density positively correlates with response to immune checkpoint blockade, suggesting that assessing their presence, and potentially their expression of inhibitory ligands such as PD-L1, could provide valuable prognostic and predictive information in immunotherapy.

Endothelial PD-L1 provides a strong rationale for combining anti-angiogenic agents with PD-1/PD-L1-targeting antibodies. VEGF has been shown to enhance the expression of several immune checkpoints including PD-1 on the surface of CD8^+^ T cells in tumors (83), while inducing PD-L1 expression by various cellular components of the TME including endothelial cells (84). Bevacizumab is an anti-VEGF antibody known to promote vascular normalization, improve tumor perfusion, reduce hypoxia, and enhance immune-cell infiltration. When combined with PD-1/PD-L1 blockade, Bevacizumab will improve PD-L1 targeting on endothelial, stromal, and immune cells, thereby mitigating multiple layers of immunosuppression. These combined effects are thought to underlie the therapeutic synergy observed between anti-VEGF and anti–PD-1/PD-L1 therapies (84). Translational evidence has since confirmed this concept in the clinic: in patients with unresectable Hepatocellular Carcinoma (HCC), Bevacizumab combined to Atezolizumab drastically improved overall and progression-free survival compared to Sorafenib, a tyrosine kinase inhibitor (85). The clinical success brought this combination therapy as the first line treatment for unresectable HCC (86) and NSCLC (87), and anti-VEGFR axitinib in combination with Pembrolizumab was approved in advanced RCC patients (88). Currently, next-generation molecules are being developed to simultaneously target both PD-1 and VEGF signalling by mean of bispecific antibodies. Ivonescimab (AK112), a humanized bispecific IgG1 antibody with dual PD-1, and VEGF-binding domains, has recently shown encouraging results in early-phase clinical trials for NSCLC and other solid tumors (89, 90).

In this chapter, we’ve highlighted the significance of examining PD-L1 expression not only in tumor cells but also in non-tumor cells present in the TME. While we won’t delve into specifics here, it is important to recognize that Tumor-Associated Neutrophils (91, 92) and Cancer-Associated Fibroblasts (93) are potential contributors to PD-L1-mediated immunosuppression. Considering all these cell types when assessing PD-L1 expression in the TME might enhance patient selection for immunotherapy, potentially improving the response to anti-PD-L1 treatments.

### Bringing the importance of the cellular sources of PD-L1 into the clinic - PD-L1 detection by immunohistochemistry

**Figure 6:**
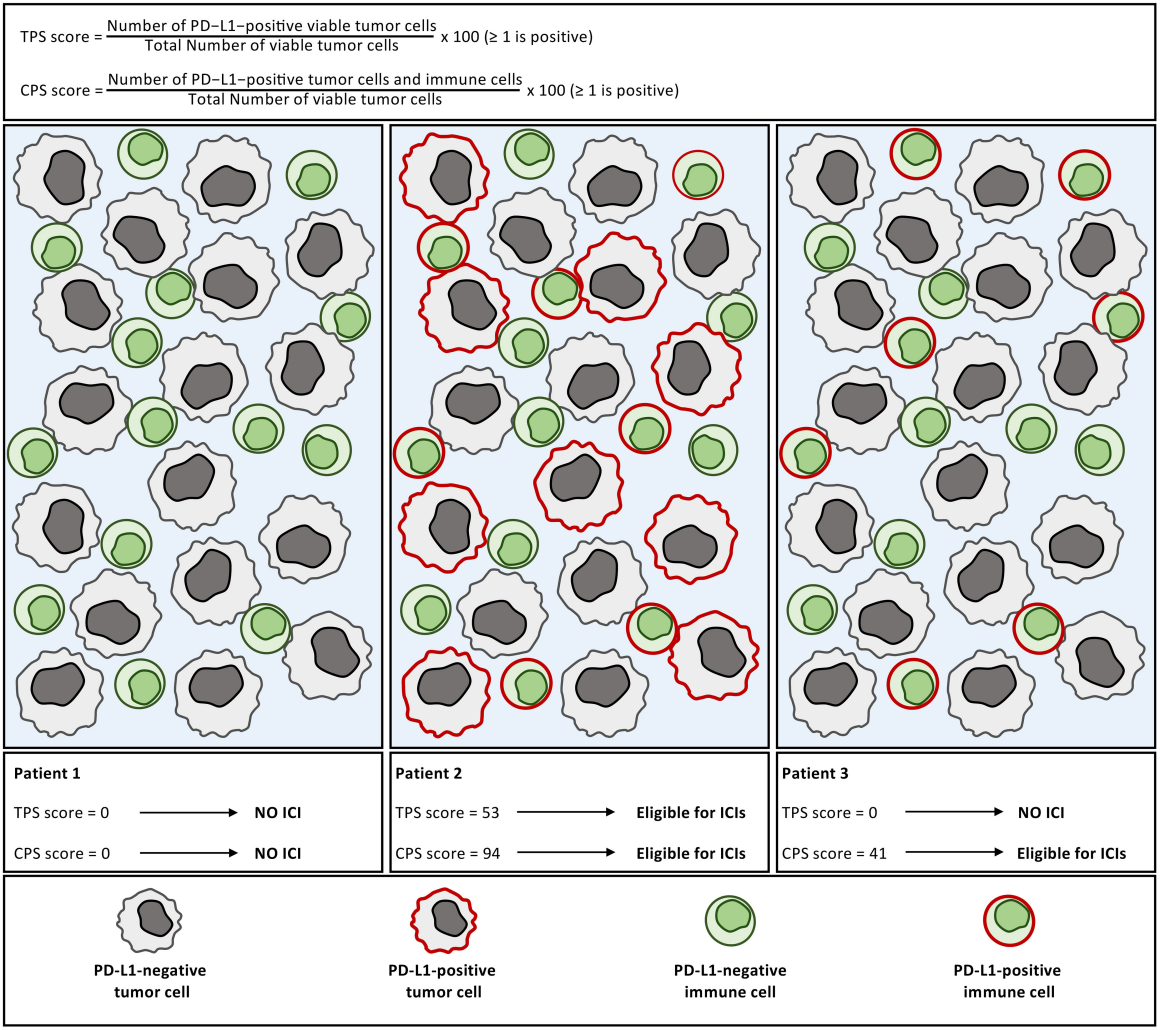
Comparison of TPS and CPS scoring systems for PD-L1 assessment in the clinic. The Tumor Proportion Score (TPS) quantifies PD-L1 expression only on tumor cells, whereas the Combined Positive Score (CPS) also includes PD-L1^+^ immune cells (mainly macrophages and T cells) identified by the pathologist.Three illustrative cases are shown: Patient 1: no PD-L1 expression (TPS = 0, CPS = 0); ineligible for pembrolizumab. Patient 2: PD-L1 expression on both tumor and immune cells; eligible for pembrolizumab under both scoring systems. Patient 3: PD-L1 expression restricted to immune cells; excluded by TPS but eligible under CPS, which better captures potential responders.

In clinical practice, anti-PD-1/PD-L1 therapies are often prescribed based on PD-L1 detection in tumor biopsies by immunohistochemistry (IHC) (**Figure 6**, **Table 1**). Pathologists typically determine a Tumor Proportion Score (TPS), which quantifies PD-L1 expression on tumor-cell membranes, while excluding immune cells that may also express PD-L1. This approach can lead to the exclusion of patients with PD-L1-negative tumors but PD-L1-positive immune infiltrates such as macrophages, DCs, or T cells populations that may in fact represent key pharmacological targets of PD-L1 blockade. The Combined Positive Score (CPS), which integrates PD-L1 expression on both tumor and immune cells (macrophages and T cells), was therefore proposed to improve predictive accuracy and correlates more consistently with responses to ICIs (94–96).

**Table 1:** Current cancer indications benefiting from TPS or CPS scoring systems for PD-L1 assessment.

Cancer type	Indication	Score and threshold
NSCLC Metastatic NSCLC	Pembrolizumab (anti-PD-1)	TPS ≥ 1
HNSCC Metastatic HNSCC	Pembrolizumab	CPS ≥ 1
Oesophageal Cancer	Pembrolizumab	CPS ≥ 1 or ≥10
Cervical cancer Metastatic Cervical cancer	Pembrolizumab	CPS ≥ 1
TBNC Metastatic TNBC	Pembrolizumab	CPS ≥ 10
Metastatic NSCLC	Atezolizumab (ani-PD-L1)	TC ≥ 50 or IC ≥ 10

Overview of tumor types in which PD-L1 expression is routinely evaluated using either the TPS or the CPS. These scoring systems guide patient eligibility for immune checkpoint inhibitors, with thresholds varying according to cancer type and therapeutic context. NB: TC (Tumor Cell score) and IC (Immune Cell score) indicate the overall percentage of the respective cell surfaces stained by anti-PD-L1 antibodies.

Nevertheless, PD-L1 IHC remains a suboptimal biomarker due to both biological and technical constraints. Mechanistically, PD-L1 expression should not be interpreted analogously to oncogenic target detection (as for HER2 or EGFR), since the therapeutic action of anti-PD-1/PD-L1 antibodies occurs primarily at the immune synapse rather than through direct elimination of PD-L1-positive tumor cells. Moreover, PD-L1 expression often mirrors an IFN-γ-driven inflamed microenvironment but may also occur constitutively in “cold” T cell-poor tumors, which rarely respond to checkpoint blockade (97). Additional variability arises from the use of distinct PD-L1 IHC antibody clones. 28-8 (used as the companion diagnostic for nivolumab), 22C3 (for pembrolizumab), SP263 (for durvalumab), and SP142 (for atezolizumab), which differ in epitope specificity and sensitivity. These discrepancies are particularly marked for immune-cell PD-L1 detection, whereas tumor-cell staining tends to be more concordant (98). Furthermore, PD-L1 glycosylation can mask epitopes, and tissue deglycosylation was shown to enhance staining intensity and improve correlation with therapeutic responses (99). Beyond reagent variability, heterogeneity in membranous versus cytoplasmic staining criteria, cell morphology interpretation, and positivity cutoffs (TPS ≥1% *vs.* ≥50%, CPS ≥1 *vs.* ≥10) generate inconsistencies across assays and complicate inter-trial comparisons. A tumor dominated by PD-L1^+^ immune cells but lacking membranous tumor staining may thus be TPS-negative yet CPS-positive, highlighting the contextual nature of PD-L1 scoring. Across cancer types, PD-L1 expression correlates with improved response rates but retains limited predictive power, with a positive predictive value often below 50% and a poor negative predictive value, indicating that PD-L1-negative patients can still respond (100). As a result, PD-L1 IHC is used as an eligibility biomarker in certain cancers (e.g., NSCLC, HNSCC, urothelial carcinoma) but not as an absolute exclusion criterion in others (e.g., melanoma). Rigid PD-L1 cutoffs may thus exclude rare but durable responders, challenging the balance between treatment access, cost, and toxicity. PD-L1 scores should therefore be viewed as imperfect surrogates of immune activation rather than measures of true target engagement. This understanding has prompted the development of multiparametric biomarkers integrating both tumor-intrinsic and immune-contextual features. PD-L1 IHC partially overlaps with T cell-inflamed gene expression profiles (GEP), whereas tumor mutational burden (TMB) captures distinct responder subsets. Combining these parameters (e.g., PD-L1 + TMB or GEP + TMB) and implementing multiplex IHC panels including PD-1, PD-L1, CD3, and CD8 could enhance predictive precision (100). Additional biomarkers, such as circulating and soluble immune factors, microbiome composition, or host genomic and metabolic signatures, have also shown promise in refining patient selection (101). Finally, integrating biomarkers predictive of immune-related adverse events may help balance efficacy and toxicity, ultimately fostering more personalized and context-aware immunotherapy strategies.

### Non-immune roles of PD-L1

**Figure 7:**
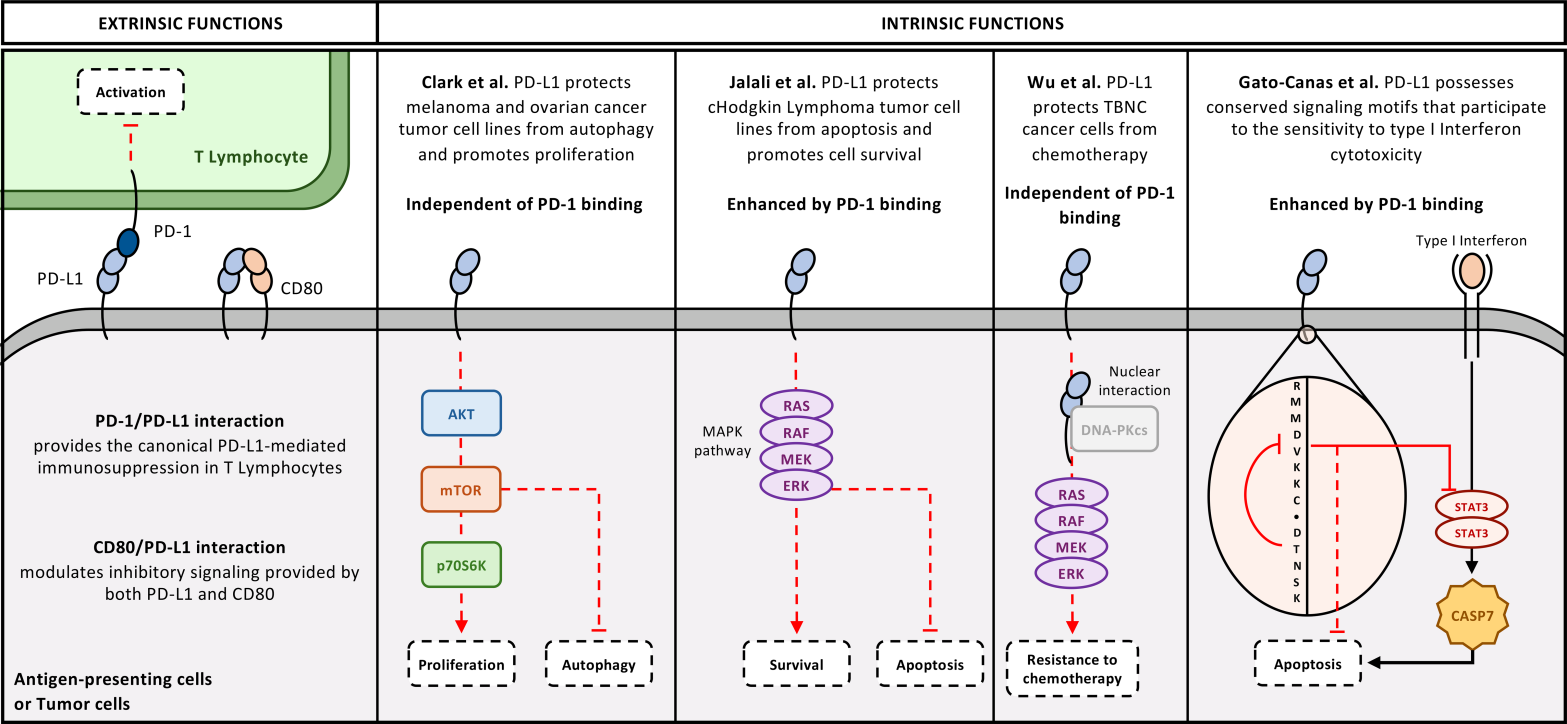
Extrinsic and Intrinsic functions of tumor-cell PD-L1. Schematic representation of the dual roles of PD-L1 in tumor and antigen-presenting cells. Extrinsic functions (left): The PD-1/PD-L1 interaction mediates the canonical inhibitory signalling that suppresses T-cell activation, while the CD80/PD-L1 *cis*-interaction modulates the strength of inhibitory signalling delivered by PD-L1 and CD80. Intrinsic functions (right): Tumor-intrinsic PD-L1 can activate or modulate multiple signalling cascades, including the AKT/mTOR/p70S6K axis promoting proliferation, the MAPK pathway enhancing survival or autophagy, and nuclear interactions with DNA-PKcs that confer resistance to DNA-damage-inducing chemotherapy. PD-L1 can also regulate apoptosis through the STAT3-CASP7 axis through conserved signalling motifs.

Most of the research on the PD-1/PD-L1 axis tackled the extrinsic functions of PD-L1 expressed by APCs and tumor cells towards PD-1-expressing cells, particularly T cells. However, shortly after the discovery of the PD-L1 protein, an increasing body of evidence suggested that PD-L1 also possessed intrinsic cellular functions (**Figure 7**). These functions depend on a wide range of parameters, such as the subcellular localization of PD-L1, the presence or absence of PD-1, as well as the integration of genetic factors and cellular pathways involved in this signalling.

Early evidence came from the research of Lieping Chen: in 2008, he and his team published a study demonstrating that PD-L1 serves as a molecular shield for tumor cells against lysis by CTLs (102). Surprisingly, this protection was not conferred by the classical PD-1/PD-L1 pathway and the associated inhibitory PD-1 signalling, but rather by PD-L1 itself, whose intracellular domain provided anti-apoptotic signals in tumor cells following engagement by PD-1. After this study, the field remained relatively quiet for a few years. Later, Clark et al. made interesting observations: firstly, they highlighted that proliferation of melanoma and ovarian cancer cells was promoted by PD-L1 expression. The use of antibodies targeting PD-L1 was able to abrogate these effects. Secondly, the use of anti-PD-L1 antibodies in immunodeficient mice bearing B16 tumors expressing endogenous levels of PD-L1 led to better control of tumor growth. RNA-sequencing analyses demonstrated that PD-L1 expression influenced various genes involved in cell survival under starvation conditions, autophagy, and mTOR-related signalling. *In vitro*, Clark et al. ultimately demonstrated that PD-L1 inhibited autophagic flux by promoting mTORC1 activity (103, 104). It is also reported that PD-L1-mediated reverse signalling in cHL cell lines increases their survival and proliferation through a process involving the MAPK signalling (105). Importantly, the latter findings were linked to PD-1/PD-L1 interaction as anti-PD-1 antibodies abrogated these pro-tumoral effects.

It is very surprising to discover that PD-L1 can be a signalling molecule considering that this protein does not possess a conserved and known motif capable of generating signalling. In 2017 and 2018, two studies provided more in-depth insight into the potential mechanisms that may directly link PD-L1 to intracellular signalling pathways. It was first shown in murine tumor cells that PD-L1 provided protection against IFN-β-induced cytotoxicity and prevented activation of the STAT3-CASPASE7 pathway. For this protection to occur, the RMLDVEKC sequence, a motif conserved across several mammalian species and present in the intracytoplasmic region of PD-L1, was necessary. Additionally, another intracellular sequence, DTSSK, serves as a regulator of the aforementioned sequence and suppresses the associated protective functions. Mutations in the DTSSK motif exist in human carcinoma cell lines, contributing to protection of tumor cells against IFN-β toxicity (106). More interestingly, these functions of PD-L1 can be enhanced by its interaction with PD-1, and the use of antibodies blocking the PD-1/PD-L1 interaction can thus reduce these effects. Afterwards, in their study attempting to understand how PD-L1 overexpression protects tumor cells from chemotherapy, Wu et al. discovered that PD-L1 can be internalized and associate with DNA-PKcs in the nucleus (107). This interaction increases ERK activation in melanoma cells and p38 MAPK pathway activation in triple-negative breast cancer (TNBC) cells and ultimately increases Survivin expression in both cases. As previously discussed, deacetylation of PD-L1 is responsible for its translocation into the nucleus (35). In this study, the authors identified by Chip-seq that PD-L1 can function as a transcriptional co-factor for the expression of different genes involved in the immune response and its regulatory pathways, including the NF-κB pathway. In this case, PD-L1 can regulate the anti-tumor immune response by promoting mechanisms of immune escape, including its own expression.

In summary, PD-L1-mediated signalling can be grouped into two main patterns. The first relies on surface PD-L1 engaging PD-1 (and possibly CD80). The second is independent of PD-1 and arises from intracellular PD-L1 pools (cytoplasmic or nuclear) (108). These non-classical layers do not replace the canonical PD-1/PD-L1 pathway but rather extend and condition it. *Cis*-interactions tune signalling at the immune synapse, intrinsic PD-L1 signalling supports tumor survival under immune pressure, and multi-cellular PD-L1 expression broadens the effects within the TME. These new insights are crucial for targeting PD-L1 protein in the context of anti-cancer immunotherapy. Indeed, the impact of therapeutic anti-PD-L1 antibodies such as Atezolizumab on these intrinsic functions of PD-L1 remains unknown, so it is unclear whether they have agonistic or antagonistic effects on PD-L1-dependent signalling pathways. Additionally, while much of the investigation in this field has focused on tumor cells, it is important to remember that other cells important in the anti-tumor immune response also express PD-L1, and it is unknown whether they are impacted in the same way by PD-L1-associated intracellular signalling. In the future, better understanding PD-L1 functions may pave the way for improved combination therapies, as exemplified by HDACi and anti-VEGF treatments (**Table 2**).

**Table 2:** Canonical and non-canonical targets modulating the PD-1/PD-L1 axis.

Pathway targeted	Target	Drug	Mechanistic rationale	Status
Canonical pathway	PD-1	Nivolumab Pembrolizumab Tislelizumab Serplulimab	→ Block inhibitory PD-1 signalling on T cells, restoring cytotoxicity	Approved for multiple cancer types
	PD-L1/PD-1 PD-L1/CD80	Durvalumab Atezolizumab Avelumab Sugemalimab	Prevent PD-L1 engagement with PD-1 and CD80 → reinvigorate T cells	Approved for multiple cancer types
Non-canonical pathway	HDAC2	Non-selective HDACi Entinostat Vorinostat Romidepsin Citarinostat Domatinostat	HDAC2 promotes PD-L1 nuclear translocation (via deacetylation) → Inhibition enhances anti-PD-1 efficacy	Clinical (Phase I–II trials in combination with anti-PD-1/PD-L1). Safety validated, but efficacy not yet demonstrated
	Cholesterol	Statins (e.g. Simvastatin, Atorvastatin)	Membrane cholesterol favors PD-L1 clustering and PD-1 engagement → Cholesterol depletion reduces PD-L1 expression and favours T-cell function	Preclinical and retrospective clinical evidence. Prospective trials ongoing
	EGFR and other RTKs	Erlotinib Gefitinib Osimertinib Cabozantinib Sunitinib Lenvatinib	Oncogenic RTK signalling (MAPK/PI3K) upregulates PD-L1 → inhibition synergizes with PD-1 blockade	Approved or in clinical trials with PD-1/PD-L1 inhibitors. Safety established; efficacy context-dependent (tumor type specific).
	VEGF(R)	Bevacizumab Ramucirumab Axitinib Lenvatinib	VEGF suppresses immune infiltration and promotes PD-L1 → anti-angiogenics normalize vasculature and improve immune access	Approved combinations with anti-PD-1/PD-L1 (e.g. Atezolizumab + Bevacizumab in HCC, Pembrolizumab + Axitinib in RCC). Clinical efficacy validated.

This table summarizes EMA-approved and emerging therapeutic targets influencing PD-1/PD-L1 signalling. Canonical targets (PD-1, PD-L1) are clinically validated checkpoint blockers. Non-canonical pathways (HDAC2, cholesterol metabolism, RTKs, VEGF) regulate PD-L1 expression, localization, or immune suppression.

While PD-L1 biology has expanded in unexpected directions, it remains inseparable from its canonical partner PD-1. We will now shift our focus to PD-1 itself, a receptor that sits at the core of immune regulation and clinical immunotherapy.

## Programmed cell death 1: a protein at the core of conventional immunotherapy

### PD-1, a master regulator of autoimmunity

The PD-1 gene and its protein product was the focus of extensive research, with T. Honjo and his collaborators as the pioneers of the early days of PD-1. Indeed, for about 10 years, several studies have been published by this group and demonstrated the crucial roles of PD-1 in immune regulation and autoimmune diseases. Soon after PD-1 discovery, in 1994, Shinohara et al. detailed the structure and chromosomal localization of the human PD-1 gene, providing a foundational understanding of its genetic makeup and positioning on chromosome 2 (2). This discovery set the stage for subsequent research into the gene’s functional implications. Agata et al. further explored PD-1 by examining its expression on the surface of stimulated mouse T and B cells (109). They found that PD-1 was upregulated upon cell activation (as opposed to its initially attributed role in cell death), suggesting a role in the regulation of immune responses. Concurrently, Nishimura et al. observed that the PD-1 protein was expressed on double-negative (CD4^-^CD8^-^) thymocytes, indicating a developmentally regulated pattern of expression and hinting at a role in thymocyte development (110). Building on these findings, studies on PD-1 deficient mice brought tremendous insight in the understanding of PD-1 functions, and demonstrated that the absence of PD-1 led to hyperactive B cell responses, implying that PD-1 acts as a negative regulator in the immune system (111). This regulatory role was underscored by subsequent research in 1999, where Nishimura et al. showed that disruption of the PD-1 gene caused mice to develop lupus-like autoimmune diseases (112). Further investigations revealed that PD-1 deficiency could lead to autoimmune dilated cardiomyopathy, a severe heart condition (113). These studies highlighted the critical function of PD-1 in maintaining self-tolerance and preventing autoimmunity. Mechanistically speaking, Okazaki et al. discovered that PD-1 inhibits B cell receptor-mediated signalling by recruiting SHP-2, a phosphatase, to dephosphorylate signalling molecules, elucidating the molecular mechanisms behind PD-1’s inhibitory functions (114). Ansari et al. found that the PD-1 pathway plays a protective role against autoimmune diabetes in nonobese diabetic (NOD) mice, as PD-1 deficiency led to disease acceleration. This highlights its relevance as a potential therapeutic target for preventing or delaying type 1 diabetes (115). Lastly, Arlene Sharpe and her team demonstrated that interactions between PD-1 and its ligand PD-L1 inhibit TCR-mediated positive selection of thymocytes, highlighting another mechanism by which PD-1 maintains immune homeostasis and prevents the development of autoimmunity (116).

### PD-1 expression and signalling in T cells

**Figure 8:**
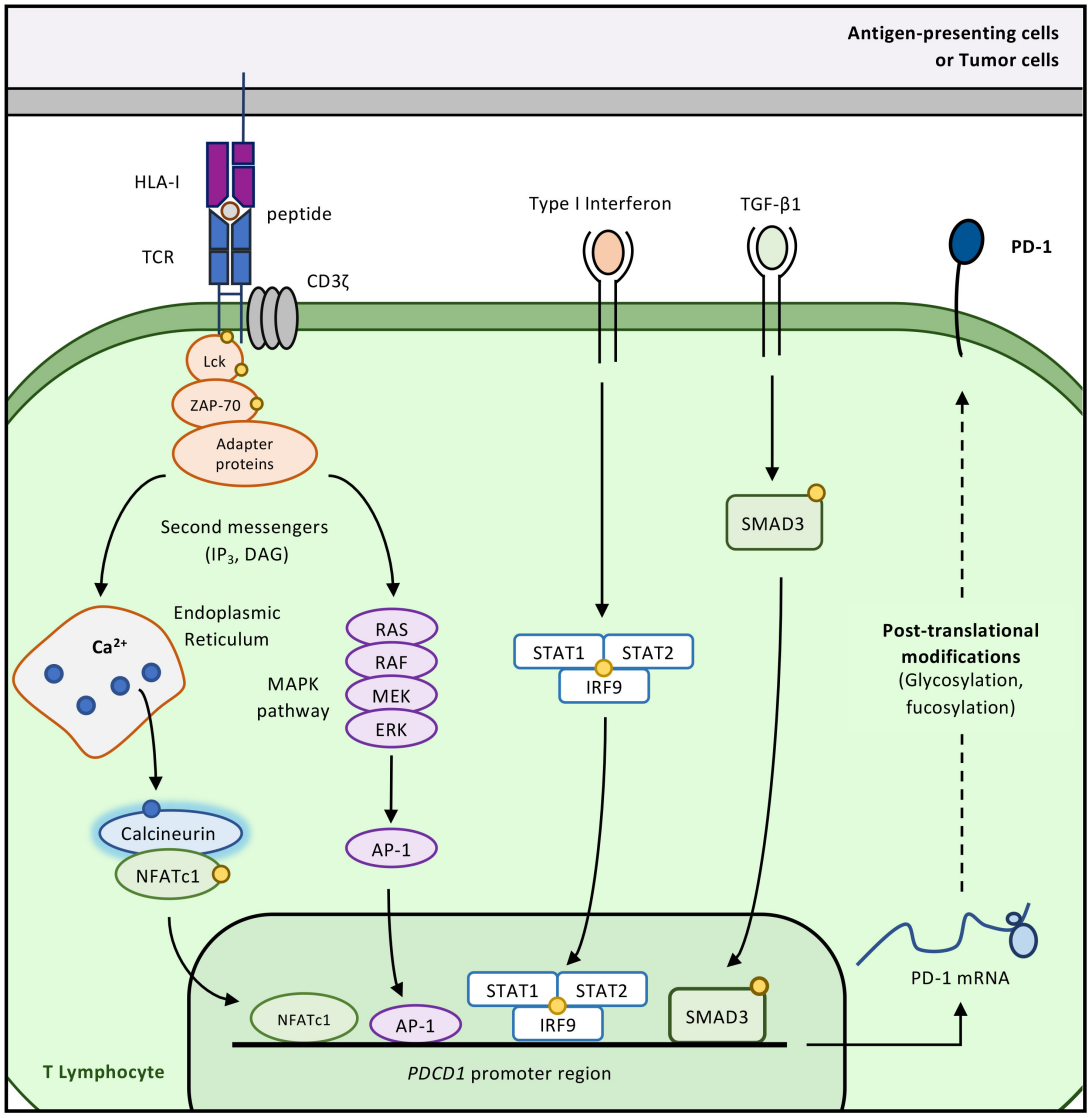
Regulatory mechanisms of PD-1 expression. Upon TCR engagement, the phosphorylation cascade involving LCK, ZAP70 and PLC-y ultimately leads to the production of inositol-3-phosphate (IP3) and diacylglycerol (DAG) from phosphatidylinositol 4,5- bisphosphate (PIP2). Upon receptor (IP3R) binding on the endoplasmic reticulum (ER), IP3 induces the release of ER calcium stores into the cytoplasm. Rapid increase of cytoplasmic calcium is responsible for the activation of calcineurin which in turns drives the nuclear translocation of the nuclear factor of activated T cells (NFAT). It has been shown *in vitro* and *in vivo* that NFAT binds to *cis* regulatory elements in the PD-1 gene (*PDCD1*) and regulates the latter transcription. As NFAT forms complexes with AP-1 transcription factors, it appears that the activation of the MAPK pathway (driven by DAG production upon TCR activation) is also an important mediator of PD-1 expression. As it was the case for PD-L1, inflammatory cues also regulate PD-1 expression. Indeed, it has been shown that the 5’-flanking region of the PD-1 gene was composed of conserved and putative IFN-stimulated response element (ISRE), allowing the binding of Interferon Responsive Factors (IRFs). Of note, type I IFN (IFN-α and IFN-β) are potent positive regulators of PD-1 expression. Moreover, common γ-chain cytokines (IL-2, IL-7, IL-15, and IL-21) can upregulate PD-1 expression in T cells. Lastly, TGF-β, by inducing SMAD3 phosphorylation, as been shown as a potent inducer of PD-1 expression in T cells.

As stated above, PD-1 expression is triggered by TCR or B-cell Receptor activation following antigen recognition, as further described in **Figure 8** (117–120).

In order to be expressed and stabilized at the plasma membrane, PD-1 undergoes PTMs that prevent the protein from being degraded. The extracellular domain of PD-1 is heavily glycosylated, at four different sites: N49, N58 (N54 in mouse PD-1), N74, and N116 (121) (**Figure 2**, Top). Interestingly, the glycan structures of PD-1 are importantly modified upon TCR activation as the latter globally increases the glycosylated forms of PD-1 within T cells. If PD-1 is experimentally deglycosylated, its stability and membrane expression are rapidly lost, with the expected consequence that it decreases its ability to bind to its ligands. One of the important glycosylation pattern of PD-1 is the addition of fucose sugars (fucosylation) on two of its putative glycosylation sites, namely N49 and N74 by a core fucosylation enzyme, Fut8 (122). However, compared to general glycosylation, it is unclear how fucosylation affects PD-1 stability at the plasma membrane, although it has been shown that loss of this modification alters PD-1 function and enhances T-cell activation. In chronically stimulated T cells, PD-1 surface expression may depend more on glycosylation and fucosylation than on ongoing transcription, underscoring the importance of these PTMs (123).

PD-1 shares the basic structure of B7-family molecules and consists of a C-terminal intracellular domain that mediates signalling, a transmembrane domain, and an immunoglobulin variable-type (IgV) extracellular domain for ligand binding (**Figure 2**, Top). PD-1 is traditionally thought to function as a monomeric receptor at the plasma membrane, though recent findings suggest that it can form homodimers via its transmembrane domain (124). The intracellular domain of PD-1 contains two highly conserved motifs: the immunoreceptor tyrosine-based inhibitory motif (ITIM) and the immunoreceptor tyrosine-based switch motif (ITSM). Upon ligand binding, PD-1 relocates to the plasma membrane near the TCR, forming microclusters (125). This proximity allows kinases downstream of TCR signalling to phosphorylate PD-1 on its ITIM and ITSM. Phosphorylated PD-1 transiently interacts with SH2-containing signalling molecules, notably SHP-2, which is the main driver of PD-1 signalling (114, 125, 126). In line with the ability of PD-1 to cluster with the TCR, it has been shown that PD-1 provides negative cooperativity for antigen recognition by disrupting the trimolecular complexes formed by the TCR, CD8 and peptide-HLA (127).

**Figure 9:**
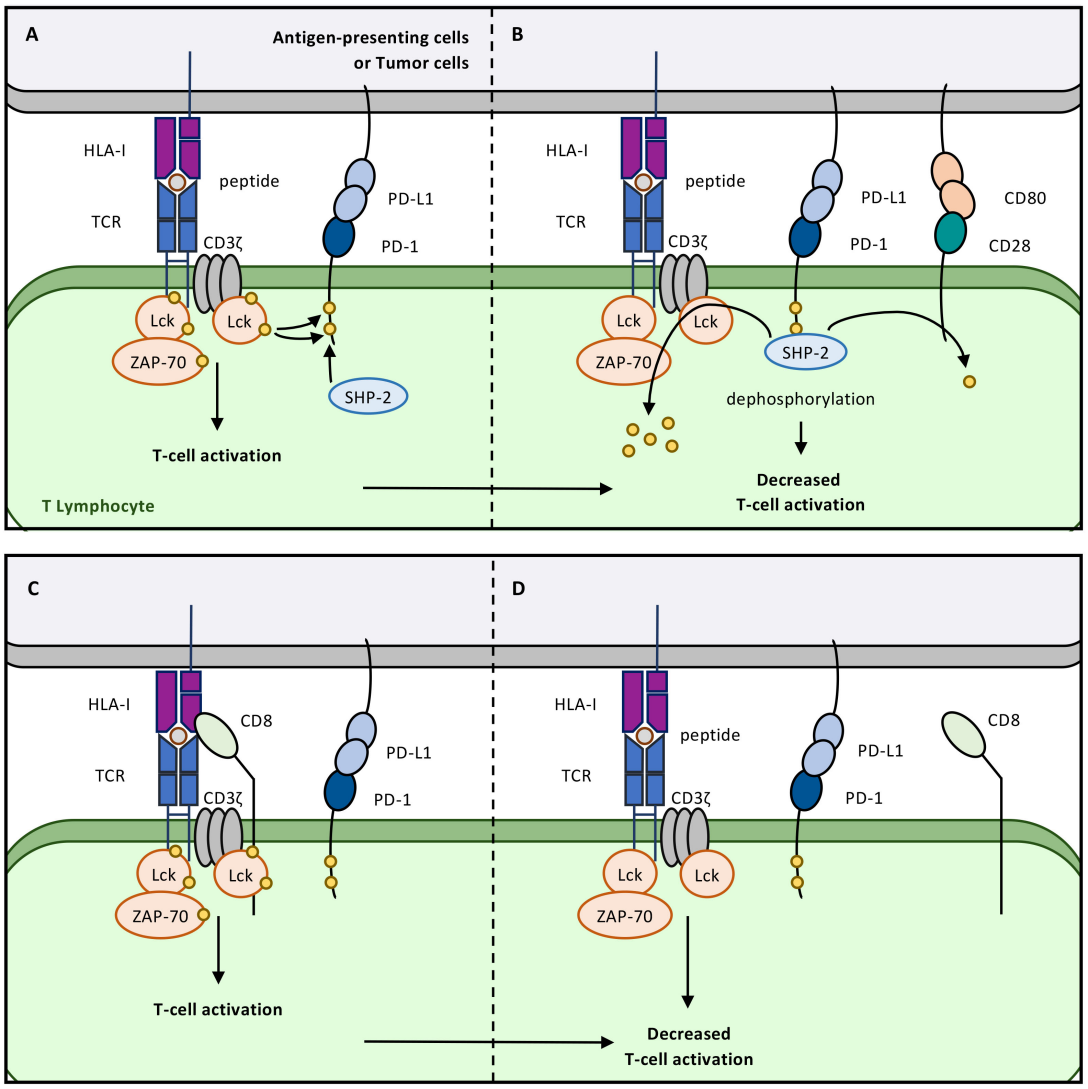
PD-1 signalling. **(A)** Upon binding of an HLA-presented peptide to the TCR, protein tyrosine kinases such as Lck will phosphorylate notably CD3ζ chains and ZAP-70 kinase in T cell. These phosphorylations are followed by intracellular signalling triggering T-cell activation. If PD-L1 is present on target cell, PD-1 is recruited to join TCR microclusters and gets phosphorylated by Lck on its ITIM and ITSM domains. Src homology region 2 domain-containing phosphatase-2 (SHP-2) is then recruited to the phosphorylated ITSM domain of PD-1. **(B)** Activated SHP-2 phosphatase dephosphorylates the transducers of the TCR activation pathway, such as Lck, ZAP-70 or CD3ζ chains. If the target cell expresses CD80, CD28 on T cells gets phosphorylated and some have suggested that CD28 might be the preferential target of SHP-2 upon PD-1 activation. **(C)** TCR and CD8 cooperate for antigen recognition and TCR activation. On one hand, CD8 stabilizes TCR-peptide-HLA recognition, on the other hand it keeps Lck kinase close to the TCR, enabling the phosphorylation of the TCR machinery components. **(D)** In presence of PD-1/PD-L1 interaction, PD-1 disrupts the trimolecular complex formed by CD8, the TCR and HLA-peptide complex, therefore preventing antigen recognition and TCR activation.

Initially, it was believed that PD-1, through SHP-2 activation, primarily targeted the TCR signalling cascade and indirectly affected CD28-mediated signalling. Indeed, Yokosuka et al. demonstrated that PD-1 engagement led to the dephosphorylation of CD3-ζ, PLCγ1, and ERK (components of TCR signalling) and disrupted the interaction between CD28 and PKC-θ (125). However, in 2017, Hui et al. revealed the significant impact of PD-1 signalling on CD28 costimulation (128). Using large unilamellar vesicles (LUVs) that mimic the T-cell plasma membrane and the immunological synapse, they showed that CD28-associated phosphotyrosine was more sensitive to PD-1-mediated dephosphorylation than other TCR signalling mediators. In cellular models, this is evidenced by the formation of microclusters between PD-1 and CD28 upon immunological synapse formation, with PD-1 segregating more with CD28 than with the TCR (**Figure 9**). These findings suggest that PD-1/PD-L1 blockade mainly acts at T cell-APC interfaces expressing CD80, rather than at T cell-tumor cell contacts. The full potency of PD-1 signalling is likely exploited when both TCR engagement (signal 1) and CD28 engagement (signal 2) are present. Since most tumor cells do not express CD80 or CD86, effective PD-1 signalling occurs mainly at the interface between T cells and APCs.

As PD-1 signalling targets early events of T-cell activation, its consequences are likely to impact multiple processes within T cells. First, PD-1 regulates several components of the cell cycle machinery. The study of Pastoukis et al. showed that, beyond inhibiting MAPK, PD-1 also prevents cyclin-dependent kinase-2 (CDK2) activation and G1–S progression by blocking the SKP2 upregulation normally induced during T-cell activation (129). It must be considered that all these effects can be partially overcome by IL-2. Furthermore, Karwacz et al. showed that PD-1 engagement participates to the expression of Casitas B‐lymphoma (Cbl)‐b E3 ubiquitin ligase, which down-modulates TCR expression at the plasma membrane (130). Of note, the latter mechanism can be efficiently abrogated by anti-PD-1 antibodies. Lastly, it is now understood that PD-1 signalling influences T-cell metabolism. The study of Patsoukis et al. in 2015 highlighted that PD-1 engagement by PD-L1 reduces both glucose consumption and utilization as well as the intake of amino acids such as glutamine and branched amino acids and their metabolism (131). This induces a switch on T-cell metabolism as exemplified by the upregulation of lipolysis and fatty acid β-oxidation (FAO) of endogenous lipids through persistent increase of CPT1A expression, the rate-limiting enzyme of mitochondrial FAO. This provides a mechanism by which glycolytic effector T cells are converted into memory T cells that rather rely on FAO for energy supply to prolong their survival.

### PD-1 targeting in cancer

PD-1 expression by TILs is a key rationale for using anti-PD-1 monoclonal antibodies in cancer treatment (132). This PD-1 expression is thought to result from the chronic stimulation of their TCR by persistent exposure to specific antigens. These TILs share similar features with T cells continuously exposed to viruses, a phenomenon extensively studied in mice with lymphocytic choriomeningitis mammarenavirus infections (LCMV) (132, 133). From these studies, the concept of exhausted CD8^+^ T cells emerged. Exhausted CD8^+^ T cells are characterized by impaired functionality, high PD-1 expression and other immune-regulatory receptors, and a distinct transcriptional program driven by transcription factors such as TOX, TCF-1, Eomes, and T-bet. In recent years, several subsets of these exhausted CD8^+^ T cells have been identified and classified based on surface marker expression. Progenitor exhausted CD8^+^ T cells express high levels of Ly108, a surrogate for TCF-1 expression, while intermediate and terminally exhausted CD8^+^ T cells do not (134). PD-1 expression varies across these subsets, with terminally exhausted CD8^+^ T cells exhibiting the highest levels.

In murine cancer models, treatments targeting the PD-1/PD-L1 axis have been shown to particularly affect the populations of TCF-1^+^, precursor exhausted T cells within the tumor bed (135, 136). These treatments induce their development and differentiation into effector cells and increase their pool within the tumors (137–139). These findings are consistent with those observed in models of chronic LCMV infection (140). In humans, the presence of PD-1^+^TCF-1^+^ TILs serves as a favourable predictive factor for anti-PD-1 therapy (135, 138). Most CD8^+^ T cell clonotypes found in tumors after therapy are already present before treatment. However, some studies also detect new clonotypes after anti-PD-1, both in the TME and in patients’ blood (141–143). These new CD8^+^ T cell clones may originate from peripheral activation in secondary lymphoid organs, but it is also possible that they come from intratumoral immune niches composed of APCs (144).

### The hidden faces of PD-1: non-T cell PD-1 expression

#### Natural killer cells

Natural Killer (NK) cell-expressed PD-1, when engaged by its ligands, can inhibit NK-cell functions such as cytotoxicity and cytokine production, similar to its effects on T cells. This inhibition can reduce the ability of NK cells to kill tumor cells and thus may contribute to immune evasion by the tumor. This is of particular interest in patients where tumors tend to evade the immune system by dampening antigen presentation by HLA-I molecules. In these cases, as it is observed in cHL, NK cells become an efficient weapon against tumor cells, again supporting the non-neglectable benefits of PD-1/PD-L1-targeting therapies in cHL patients (145).

While the mechanisms of PD-1 expression on T cells, which is linked to TCR engagement, are well understood, recent research has begun to unravel how PD-1 is induced on NK cells. It has been observed that resting NK cells contain PD-1 within intracellular compartments, particularly in the Golgi, suggesting that PD-1 can be rapidly expressed on the cell surface in response to certain stimuli (146). Glucocorticoids (GCs) have been identified as crucial for inducing PD-1 expression on NK cells in both mice and humans (147). In mouse models, GCs were shown to induce PD-1 expression on NK cells during viral infections, which in turn inhibited the production of IFN-γ, thus protecting the host from the harmful effects of hyperinflammation. However, there are notable differences between species. In human NK cells, PD-1 induction takes longer and requires additional cytokines, such as IL-12, which is not necessary in mice (148). The clinical implications of these findings are significant, particularly concerning cancer treatment. GCs are frequently administered to cancer patients to manage the side effects of immunotherapy. However, GCs may also induce PD-1 expression on NK cells, potentially diminishing the effectiveness of PD-1/PD-L1-targeted immunotherapies. This is because GCs can induce PD-1 expression on NK cells, increasing their susceptibility to inhibition by PD-L1 in the TME, thereby counteracting the intended effect of immune checkpoint blockade. Studies in lung cancer patients (NSCLC) suggest that corticosteroid treatment can lead to worse outcomes when combined with PD-1/PD-L1 inhibitors (149).

The study by Hasim et al. (2022) introduced a novel mechanism of immune suppression in the TME, where NK cells acquire PD-1 through trogocytosis from PD-1-expressing cells (150). This process leads to the functional inhibition of NK cells, as the trogocytosed PD-1 can engage with PD-L1/PD-L2 on tumor cells, suppressing NK-cell cytotoxicity. The research demonstrates that this PD-1 acquisition impairs NK cell-mediated tumor control *in vivo*, highlighting a potential immune evasion strategy by tumors.

#### Myeloid cells

During the studies that led to the identification of PD-1, it was quickly shown that this protein is also expressed by myeloid cells, especially when these cells are activated. The role of PD-1 in myeloid cells was first explored in the context of bacterial or viral infections. In their 2009 report on resistance to Listeria monocytogenes (LM) infections, Yao et al. discovered that DCs in the spleen during LM infection expressed PD-1 (151). These findings were replicated *in vitro* by incubating freshly isolated DCs with LM. Notably, this study also demonstrated that *in vitro* stimulation with various inflammatory signals (TLR-ligands and inflammatory cytokines like IL-2, IL-12, and IFN-γ) increased PD-1 expression on activated DCs, which directly inhibited their cytokine production (IL-12 and TNF-α) in response to LM infection. Importantly, the use of antibodies that block the PD-1/PD-L1 interaction can reverse these effects. Similarly, Huang et al. made comparable observations (152). Their study found that *PDCD1^KO^* mice were less susceptible to experimental sepsis compared to wild-type mice. Their research concluded that the high expression of PD-1 by macrophages during sepsis is a marker of their dysfunction. PD-1 expression appears to be crucial for maintaining a balance between the immune response against pathogens and the associated inflammatory signals, which can sometimes be pathological. During chronic viral infection, it was also shown that PD-1 expression increased on the surface of monocytes, driven by the presence of TLR ligands and inflammatory cytokines (IL-6, TNF-α) (153). Activation of PD-1 on monocytes, either through agonistic antibodies or binding by PD-L1 (but not PD-L2), leads to increased IL-10 production by these cells. This, in turn, inhibits the proliferation and cytokine production of CD4^+^ T cells, reducing their ability to control chronic viral infections.

In the context of cancer, it has long been established that tumor-infiltrating DCs characterized by an immunosuppressive phenotype within the ovarian TME express PD-1, while secreting cytokines such as IL-6, IL-10, and G-CSF (154–157). Inhibiting PD-1 has been shown to boost inflammatory cytokine production, likely through the reactivation of the NF-κB transcription factor, which is suppressed by PD-1 signalling. Research also indicates that the loss of PD-1 expression on DCs increases their longevity within lymph nodes. Normally, PD-1 expression following DCs activation leads to their rapid apoptosis through interaction with PD-L1. Knocking out PD-1 in these cells improves antigen-specific T-cell responses, enhancing both the proliferation and cytokine production of CD4^+^ and CD8^+^ T cells. In Chronic Lymphocytic Leukemia (CLL), monocytes exhibit high levels of PD-1, which disrupts their glucose metabolism and weakens their immune functions, fostering an immunosuppressive environment that promotes leukemia progression (158). Similarly, PD-1 expression on TAMs significantly impairs their phagocytic capabilities, compromising the immune system’s ability to eliminate tumor cells and facilitating tumor immune evasion (159). Therapeutic interventions, such as anti-PD-1 therapy, have shown promise in reprogramming macrophages from an immunosuppressive M2-like phenotype to a pro-inflammatory M1-like phenotype, as demonstrated in a 2018 study on osteosarcoma (OS) lung metastases (160). This reprogramming resulted in significant tumor regression, highlighting the dual impact of PD-1 blockade, not only enhancing T-cell activity but also modulating macrophage function to strengthen anti-tumor immunity.

In 2020, Strauss and colleagues explored the effects of specifically deleting PD-1 in myeloid cells, including macrophages and DCs (161). The targeted deletion led to a substantial increase in anti-tumor immunity, underscoring the critical role of PD-1 expression in myeloid cells in suppressing immune responses against tumors. More recently, it was demonstrated that tumor-PD-L1 engaged PD-1 on myeloid cells to suppress type I interferon production, which in turn impaired the recruitment of CTLs to the tumor site (162). This suppression weakens the overall immune response against the tumor, further emphasizing the need to disrupt the PD-1/PD-L1 interaction in myeloid cells to enhance CTL recruitment and improve anti-tumor immunity. Collectively, these studies underscore the central role of the PD-1/PD-L1 axis in modulating immune functions within myeloid cells, particularly in dampening immune responses and promoting tumor progression. By blocking this pathway, it is possible to reprogram myeloid cells from an immunosuppressive to a anti-tumor phenotype, thereby enhancing the effectiveness of cancer therapies.

#### Tumor cells

In 2010, Schatton et al. made a surprising observation in the study of cellular subpopulations responsible for melanoma induction and the tumorigenic capabilities of these cells (163). They found that malignant melanoma-initiating cells expressing the chemotherapy resistance mediator ABCB5 tended to express inhibitory molecules, particularly PD-1. These cells appeared capable of inhibiting IL-2-dependent T-cell proliferation and inducing the differentiation of Tregs within the TME. In 2015, the same team repeated their observations in murine tumor models, focusing now on the functional roles of PD-1 presence on the surface of tumor cells (164). They discovered that inhibiting PD-1 expression in melanoma cells using shRNA technology significantly slowed tumor-cell growth *in vivo*. Conversely, PD-1 overexpression notably accelerated the growth of these tumor cells. Importantly, these effects seemed independent of the adaptive immune system of the mice, as the same results were observed in immunodeficient (NSG) mice and *in vitro*. PD-1-driven tumor growth seemed to require mTOR activation, PD-1 engagement by PD-L1, and intact ITIM and ITSM motifs in the PD-1 cytoplasmic tail. Interestingly, Kleffel et al. also demonstrated that antibodies targeting PD-1 in the context of immunotherapy could directly inhibit the growth of tumor cells expressing the target, even in immunodeficient models (164). In glioblastoma, Mirzaei et al. identified PD-1 expression on the surface of brain tumor-initiating cells (BTICs), although some of their observations did not fully align with Kleffel et al.’s data (165). In BTICs, PD-1-mediated tumor growth seemed independent of its interaction with PD-L1, rendering conventional therapies targeting the PD-1/PD-L1 axis ineffective. Instead, the mechanism appeared to rely on the NF-κB pathway. Finally, in a study published in 2023, Rotolo et al. confirmed the pro-tumoral role of PD-1 in tumor cells based on *in vitro* tests on lung cancer cell lines (166). Again, PD-1 expression was higher in chemotherapy-resistant cells, though it remained unclear whether PD-1 directly contributed to chemoresistance. So far, the mechanisms leading to PD-1 expression by tumor cells remain unelucidated.

While most literature agrees on a pro-tumoral role for PD-1, some sources suggest otherwise, indicating that PD-1 expression might be a tumor suppressor. For instance, Wang et al. showed that PD-1 activation on lung cancer tumor cells by its ligand PD-L1 decreased the canonical activation of signalling pathways involving AKT and MAPK, effects blocked by antibodies inhibiting the PD-1/PD-L1 axis (167). These findings correlate with data from Ieranò et al. (168). No study has yet explained these discrepancies regarding the role of PD-1 expressed by tumor cells. The variability in studied cell models and the signalling pathways involved might be a line of investigation. It is also possible that these roles are modulated by the differentiation state of the tumor cells and the chemotherapeutic/radiotherapeutic treatments they undergo.

## Let’s not forget about PD-L2

Although PD-L1 has long dominated the spotlight, it is not PD-1’s only binding partner. PD-L2, often overlooked, plays complementary and sometimes distinct roles that are crucial to understand the full spectrum of PD-1 pathway regulation.

The roles of PD-L2 in the physiological and pathological mechanisms of immunity and tolerance remain debated in the literature. PD-L2, also known as B7-DC, was first described by Latchman et al. in 2001 as a protein homologous to PD-L1, sharing 38% sequence similarity with human PD-L1 (169). In this study, the authors conducted the first analysis of PD-L2 expression patterns, finding its mRNA in organs similar to those where PD-L1 is expressed, such as the heart in humans. Notably, unlike PD-L1, PD-L2 is expressed in the pancreas, lungs, and liver in humans. At the cellular level, PD-L2 expression appears more restricted than PD-L1, being primarily found in immune cells, although it can also be observed on endothelial and epithelial cells, as well as certain tumor cells. Like PD-L1, *in vitro* binding of PD-L2 to PD-1 on activated lymphocytes triggers a signalling cascade that counteracts TCR (or BCR) and CD28 signalling, leading to reduced lymphocyte proliferation and cytokine secretion. The interaction surface between PD-L2 and PD-1 significantly overlaps with that between PD-L1 and PD-1, although some amino acids differ, and PD-L2 binds more extensively, which may explain its 2-to-6-fold higher affinity for PD-1 (170, 171). Due to its immunosuppressive functions, PD-L2 represents another explanation for the therapeutic benefit that is observed in cancer patients treated with PD-1-targetting antibodies while displaying no expression of PD-L1 in the TME.

As mentioned earlier, the roles of PD-L2 in immune regulation are still under significant debate. Early observations supporting this come from research by Tseng et al. (2001) (172) and Liu et al. (2003) (173). In their initial report on PD-L2 expression by DCs, Tseng et al. observed that *in vitro*, immobilized PD-L2 during CD4^+^ and CD8^+^ T-cell activation promoted their co-stimulation, contradicting earlier data from Latchman et al. Later, in their *in vivo* studies, Liu et al. found that genetically modified tumor cells overexpressing PD-L2 were rapidly rejected by tumor-bearing mice compared to their PD-L2-negative counterparts. Importantly, this rejection was immune-dependent, as it was not observed in *Rag*^-/-^ mice and was specifically dependent on CD8^+^ T cells, since depletion of these cells allowed PD-L2^+^ tumors to grow. *In vitro*, they demonstrated that PD-L2 on target cells enhanced the cytotoxic functions and proliferation of CTLs. The breakthrough of this study, reconciling the inhibitory and co-stimulatory roles of PD-L2, was to show that soluble PD-L2 could bind to the surface of T cells lacking PD-1 and still enhance T-cell co-stimulation. This provided the first evidence of a PD-L2 receptor other than PD-1 that produces effects opposite to those mediated by PD-1. 

Although this potential dual role of PD-L2 has not been resolved yet, research conducted by the laboratory of A. Sharpe revealed that PD-L2 interacts with a second receptor, Repulsive Guidance Molecule b (RGMb) (174). RGMs are glycosylphosphatidylinositol-anchored membrane proteins that interact with bone morphogenetic proteins (BMPs) and neogenin, acting as co-receptors to modulate BMP signalling pathways. RGMb, a member of this family, is primarily expressed in the nervous system but is also found in T cells and macrophages, though its functions in the immune system are not well understood (175). Xiao et al. demonstrated a significant inhibition of immune tolerance in an OVA-induced murine model of pulmonary inflammation by blocking the PD-L2/RGMb interaction. Interestingly, disrupting this interaction restored proliferation and cytokine production in OVA-specific CD4^+^ T cells without depleting APCs. In a subsequent study exploring this new interaction, Park et al. identified significant expression of RGMb on tumor-infiltrating CD8^+^ T cells in mouse models (176). Furthermore, treatment of these mice with antibodies specifically targeting RGMb resulted in a marked increase in CD8^+^ and CD4^+^ T-cell infiltration into the tumor bed. Their data also suggest that this treatment enhances the anti-tumor immune response. Importantly, the conditional loss of RGMb expression on T cells produced similar effects.

These findings reveal that the PD-L2/RGMb interaction acts as an immunosuppressive pathway, distinct from the classical PD-1/PD-L2 axis. This contrasts with reports indicating immunostimulatory roles for PD-L2, underscoring that PD-L2’s function is context-dependent and mediated by different binding partners that may still have to be identified. Therefore, PD-L2 can modulate immune responses either by inhibiting or enhancing activity depending on the cellular environment and receptor interactions. This duality has important implications for understanding immune tolerance and developing novel immunotherapies.

## Concluding remark

The PD-1/PD-L1 signalling pathway has become a cornerstone in the field of immunotherapy, offering a powerful tool in the battle against cancer. The introduction of checkpoint inhibitors that target this pathway has not only transformed the therapeutic landscape but has also sparked a wave of new research aimed at understanding the intricate biological mechanisms underlying its function. While the clinical success of these therapies is undeniable, the varied responses observed among patients highlight the critical need for a deeper, more nuanced understanding of the fundamental biology governing this pathway. This review has aimed to address this need by examining the PD-1/PD-L1 axis from a fundamental perspective, shedding light on key aspects that could inform and enhance therapeutic strategies.

Initially identified for their involvement in maintaining self-tolerance and preventing autoimmunity, PD-1 and PD-L1 have since been recognized as pivotal players in the immune system’s ability to respond to cancer. The development of antibodies that block the PD-1/PD-L1 interaction marked a breakthrough in oncology, allowing the reactivation of exhausted T cells within the TME and leading to significant clinical responses in various cancers. However, the heterogeneity of these responses, ranging from complete remission to partial or even absent responses, underscores the complexity of this pathway and the need for continued research into its underlying mechanisms.

PD-L1 is subjected to a variety of regulatory influences, including post-transcriptional and post-translational modifications. These mechanisms are mediated by factors such as microRNAs, epigenetic changes, and diverse signalling pathways, all of which contribute to the dynamic regulation of PD-L1 levels on the cell surface. Importantly, PD-L1 is not limited to expression on tumor cells; it is also found on a range of non-tumoral cells, including immune and stromal cells, which suggests that PD-L1 has significant roles in normal physiology, beyond its well-known function in immune evasion by tumors. Furthermore, PD-L1 has been implicated in pro-tumoral activities that are independent of its immune regulatory functions, such as promoting cell proliferation, invasion, and metastasis. These findings highlight the multifaceted nature of PD-L1 and suggest that therapies targeting this molecule could have broader implications than previously thought.

While PD-1 is critical for maintaining tolerance and preventing autoimmunity, it also plays a central role in modulating the immune response to cancer. The regulation of PD-1 expression and its downstream signalling pathways are complex and tightly controlled, involving multiple levels of regulation. In cancer, PD-1 is highly expressed on exhausted T cells, which are key targets of immunotherapy. By blocking PD-1, these therapies aim to restore the function of these T cells, as well as their proliferation, enabling them to effectively attack tumor cells. However, recent research has expanded our understanding of PD-1 beyond T cells, revealing its expression on other immune cells, such as NK cells and myeloid cells, and even on some tumor cells themselves. This broader expression pattern suggests that PD-1 may have additional roles in regulating the immune response and the TME, which could have significant implications for immunotherapy.

PD-L2, although less studied than PD-L1, emerges as an important piece of the puzzle. Its ability to bind PD-1 with greater affinity, along with the discovery of its interaction with alternative receptors like RGMb, suggests that PD-L2 could have distinct and potentially complementary roles in immune regulation. The study of PD-L2 is still in its early stages, but it is becoming clear that this molecule could influence immune responses in ways that are not entirely overlapping with PD-L1. For instance, the interaction between PD-L2 and RGMb has been shown to modulate immune tolerance in specific contexts, such as in models of lung inflammation. The fact that PD-L2 can also enhance T-cell co-stimulation independently of PD-1 hints at the existence of alternative signalling pathways that could be therapeutically exploited.

In summary, this review has sought to underscore the importance of a comprehensive understanding of the PD-1/PD-L1 signalling pathway from a fundamental perspective. The intricate regulation of PD-L1, the multifaceted roles of PD-1, and the emerging significance of PD-L2 all point to a highly complex network of interactions that govern immune responses. By deepening our knowledge of these mechanisms, we can not only improve the efficacy of current therapies but also pave the way for the development of new therapeutic strategies. Such an approach could lead to more personalized treatments, better able to account for the variability in patient responses and the complexity of tumor biology. Ultimately, integrating fundamental biology with clinical practice will be key to realizing the full potential of immunotherapy and addressing the ongoing challenges in the treatment of cancer.
